# *In silico* design and optimization of selective membranolytic anticancer peptides

**DOI:** 10.1038/s41598-019-47568-9

**Published:** 2019-08-02

**Authors:** Gisela Gabernet, Damian Gautschi, Alex T. Müller, Claudia S. Neuhaus, Lucas Armbrecht, Petra S. Dittrich, Jan A. Hiss, Gisbert Schneider

**Affiliations:** 1 Department of Chemistry and Applied Biosciences, ETH Zurich, Zurich, Switzerland; 2 Department of Biosystems Science and Engineering, ETH Zurich, Basel, Switzerland

**Keywords:** Medicinal chemistry, Protein design, Cheminformatics

## Abstract

Membranolytic anticancer peptides represent a potential strategy in the fight against cancer. However, our understanding of the underlying structure-activity relationships and the mechanisms driving their cell selectivity is still limited. We developed a computational approach as a step towards the rational design of potent and selective anticancer peptides. This machine learning model distinguishes between peptides with and without anticancer activity. This classifier was experimentally validated by synthesizing and testing a selection of 12 computationally generated peptides. In total, 83% of these predictions were correct. We then utilized an evolutionary molecular design algorithm to improve the peptide selectivity for cancer cells. This simulated molecular evolution process led to a five-fold selectivity increase with regard to human dermal microvascular endothelial cells and more than ten-fold improvement towards human erythrocytes. The results of the present study advocate for the applicability of machine learning models and evolutionary algorithms to design and optimize novel synthetic anticancer peptides with reduced hemolytic liability and increased cell-type selectivity.

## Introduction

Cancer therapy faces the challenge of resistance to chemotherapeutics and receptor-targeted anticancer agents. Several cell resistance mechanisms, such as drug inactivation or efflux, target protein alteration, DNA damage repair and signaling cascade alteration have been identified^1,2^. Moreover, the indiscriminate action of most chemotherapeutics towards all rapidly dividing cells causes a variety of severe side effects^3,4^. Membranolytic anticancer peptides (ACPs) represent a new class of potential cancer therapeutics. Their receptor-independent mechanism of action may hinder the development of cellular resistance^3–5^. Nevertheless, the underlying structure-activity relationship that explains the membranolytic properties of these peptides is not completely understood. Peptide amphipathicity, moderate overall hydrophobicity, and a positive net charge are known requirements for ACP activity^6–9^. However, no simple combination of these properties has been found sufficient to fully explain the activity and selectivity of ACPs towards cancer cells^10^. Producing novel peptides lacking toxicity against nonneoplastic cells also remains challenging^11^. Various machine learning methods have been successfully applied to guide the rational design of both ACPs^12–18^ and antimicrobial peptides (AMPs)^19,20^, as well as other membrane-active peptides^21^. The lack of a systematic annotation of the selectivity of ACPs towards cancer cells in the literature and in peptide databases has hindered the development of predictive models that take selectivity into account. There is a need for innovative methods that do not require selectivity data for peptide optimization.

Simulated molecular evolution (SME) is a stochastic optimization algorithm pioneered in the 1990s for computational peptide design^22–24^. SME belongs to the class of evolutionary algorithms, which also includes genetic algorithms, and enables the optimization of peptide properties that are encoded in a theoretical fitness function or in combination with an experimental fitness evaluation when structure-activity relationships cannot be determined *a priori*. We have recently applied this design concept to generate innovative membrane-targeting peptides^25,26^. Here, we present a peptide design approach that is based on a novel ACP prediction model and on SME for the optimization of ACP selectivity for cancer cells. The predictive machine learning model led to the discovery of four novel synthetic ACPs with low-micromolar activity (1–20 µM) against A549 lung cancer and MCF7 breast cancer cells. One of these peptides was then subjected to SME. After the first iteration of the optimization process, we obtained a novel ACP that showed micromolar activities against a range of cancer cell types with significantly reduced activity towards human dermal microvascular cells (HDMEC) and human erythrocytes. The results of this study advocate for machine-learning models in combination with computational sequence generators for designing and optimizing functional peptides *in silico*.

## Results and Discussion

### ACP classifier model

We developed a machine learning model to classify peptides into ACPs and non-ACPs based on their amino acid sequence representations. The machine-learning classifier was trained on “positive” (ACPs, active) and “negative” (non-ACPs, inactive) peptides. We retrieved alpha-helical ACPs from the CancerPPD database^27^ as positive examples (*N* = 339). For the negative class, we retrieved alpha-helices from nontransmembrane proteins in the PDB database^28^ (*N* = 680). All amino acid sequences were represented numerically in a computer-readable form by the use of molecular descriptors. For this purpose, we utilized a combination of PEPCATS pharmacophore feature descriptors^29^ and four global properties, namely, Eisenberg’s hydrophobicity, Eisenberg’s hydrophobic moment^30^, charge density, and peptide length (number of residues). The PEPCATS descriptor represents the amino acid sequences as binary vectors indicating cross-correlated pharmacophore features of the individual amino acids (hydrophobic, aromatic, hydrogen-bond acceptor, hydrogen-bond donor, positively ionizable, negatively ionizable). The cross-correlation of pharmacophoric feature pairs is determined within a sliding sequence window encompassing seven residues. The resulting 151-dimensional descriptor vector was reduced to an 18-dimensional feature vector by covariance elimination and sequential feature elimination (Fig. S1, Supplementary Information). The dataset was split into a training set (2/3) and an independent test set (1/3) by stratified sampling, preserving the proportion between the positive and negative classes. Two machine learning algorithms were considered for model development: random forests^31^ and support vector machines (SVM)^32^. We optimized the SVM model’s hyperparameter by 10-fold cross-validation on the training data and chose a linear kernel for SVM training to enable straightforward feature interpretation. The performance of both classifiers exceeded 0.9 for both the training and the test data for all calculated metrics (Table 1). The SVM model was selected for further analysis due to the robustness of its decision function, which is determined solely by the support vectors and therefore unaltered by the addition of new data points that lie outside the decision margin^32^. Additionally, an analytical decision function as a linear combination of the model features can be extracted from linear support vector machines, whose weights indicate feature importance for the classification problem^32^.

**Table 1:** Performance of support vector machine and random forest models for ACP prediction.

Metrics	Support Vector Machine			Random Forest		
	CV score	Train score	Test score	CV score	Train score	Test score
MCC	0.88 ± 0.05	0.91	0.90	0.90 ± 0.05	1	0.91
Accuracy	0.94 ± 0.02	0.96	0.96	0.95 ± 0.02	1	0.96
Precision	0.89 ± 0.04	0.92	0.91	0.96 ± 0.03	1	0.97
Recall	0.95 ± 0.06	0.96	0.95	0.90 ± 0.06	1	0.90

Scores obtained from ten-fold cross-validation (CV) score (*mean* ± *std*), on the whole training dataset (Train score) and the independent test dataset (Test score) for the support vector machine and random forest models.

We then compared the performance on the test dataset for our SVM model to online available ACP prediction tools, specifically the AntiCP models 1 and 2^13^, the iACP model^33^, and the MLACP model^18^. These ACP prediction models are also based on an SVM classifier but utilize different descriptors and training data (Table S1, Supplementary Information). The prediction performance of the four classifiers and our SVM model was assessed on the independent test dataset (Table 2). In this experiment, the performance of our SVM model on the independent test set was superior to all four publicly available ACP prediction models in terms of all performance metrics, except for precision. The MLACP model showed higher precision but lower Matthews correlation coefficient (MCC), accuracy and recall than the other models. Therefore, the MLACP model is better at avoiding false positives but retrieves a higher number of false negatives compared to the SVM model developed in this study.

**Table 2:** Comparison of the model performance (Test score) with other online available ACP prediction tools calculated by using the independent test set. The Matthews correlation coefficient (MCC), accuracy, precision and recall were used as metrics (Methods Eqs 1–4).

Metrics	AntiCP Model 1	AntiCP Model 2	iACP	MLACP
MCC	−0.04	0.81	0.51	0.84
Accuracy	0.29	0.92	0.77	0.93
Precision	0.29	0.81	0.58	0.96
Recall	0.99	0.92	0.78	0.80

### Feature importance for ACP activity

We analyzed the feature weights of the SVM classifier to gain an understanding of important discriminatory features for distinguishing between ACPs and non-ACPs (Table 3, Fig. S2, Supplementary Information). Features were ranked by their absolute weight values as a measure of their relative importance for ACP classification. The global hydrophobicity (H), hydrophobic moment (µ_H_) and the frequency of positively charged amino acid pairs separated by one residue (PPd2) were identified as important features of the classifier (weight values *w* = 1.65, *w* = 0.5 and *w* = 0.39, respectively). This finding is in accordance with previous reports on ACPs that highlight the relevance of the hydrophobicity, the hydrophobic moment and a net positive charge for anticancer activity^7,34^. The peptide length was also identified as a discriminatory feature (*w* = 0.4), indicating that longer peptides were considered more likely to be active. Two features that take into account the frequency of amino acids with hydrogen-bond donor and acceptor groups (ADd0, DDd0) were identified as bearing the greatest absolute weights (*w* = −1.94 and *w* = 1.67, respectively), emphasizing their role in distinguishing ACPs from inactive peptides (Table 3).

**Table 3:** The 18 features obtained after covariance elimination and sequential feature selection.

Feature	Weight	Description
ADd0	−1.94	Frequency of amino acids with hydrogen-bond acceptor and donor groups (T, C, Q, N, S and Y)
DDd0	1.67	Frequency of amino acids with hydrogen-bond donor groups (K, T, C, Q, H, R, W, N, S and Y)
H	1.65	Global peptide hydrophobicity (Eisenberg consensus scale^30^)
RPd0	−0.72	Frequency of aromatic amino acids with a positively ionizable group (H)
ADd2	0.65	Frequency of amino acids with hydrogen-bond acceptor and amino acids with donor groups at distance 2
µ_H_	0.50	Peptide hydrophobic moment
LDd0	0.40	Frequency of lipophilic amino acids with hydrogen-bond donor groups
Len	0.40	Peptide length
PPd2	0.39	Frequency of amino acids with positively ionizable groups at distance 2
RPd5	0.38	Frequency of aromatic amino acids and amino acids with positively ionizable groups at distance 5
APd6	−0.38	Frequency of amino acids with hydrogen-bond acceptor groups and amino acids with positively ionizable groups at distance 6
RAd3	−0.26	Frequency of amino acids with hydrogen-bond acceptor and amino acids with donor groups at distance 3
RAd2	−0.25	Frequency of amino acids with hydrogen-bond acceptor and amino acids with donor groups at distance 2
APd1	−0.25	Frequency of amino acids with hydrogen-bond acceptor groups and amino acids with positively ionizable groups at distance 1
DNd1	−0.16	Frequency of amino acids with hydrogen-bond donor groups and amino acids with negatively ionizable groups at distance 1
APd2	−0.11	Frequency of amino acids with hydrogen-bond acceptor groups and amino acids with positively ionizable groups at distance 2
RPd2	−0.08	Frequency of aromatic amino acids and amino acids with positively ionizable groups at distance 2
RRd6	0.02	Frequency of aromatic amino acids at distance 6

The top scoring features are ranked by their absolute support vector machine weight values, as a measure of their relative importance for ACP classification. An interpretation of each feature is provided.

### *De novo* design of ACPs

To make use of the SVM model for the *in silico* design of novel ACPs, we generated three virtual peptide libraries of 100,000 peptides each, based on different design principles (Fig. S3, Supplementary Information):

1. The *Helical* library contains peptides with the position-dependent amino acid distribution of alpha-helical ACPs^11^.
2. The *Amphipathic Arc* library contains amphipathic peptides with differently sized hydrophobic arcs and a high probability of being cationic.
3. The *Gradient* library contains amphipathic peptides that possess a linear hydrophobic gradient.

We predicted the activity of the peptides from each library with our SVM model (Fig. S4, Supplementary Information). More than 80% of the peptides from the *Amphipathic Arc* and *Gradient* libraries and more than 60% of the peptides from the *Helical* library received an SVM score >0.5, indicating potential actives. In contrast, only 10% of peptides with random sequences were predicted to be active. The design principles, therefore, enriched the libraries with potentially active peptides in contrast with random peptide generation.

The similarity of the peptides in the training data was analyzed to consider the applicability domain of the SVM model^35^; this domain is the chemical space in which the model predictions may be considered reliable. The SVM model was utilized to estimate the *pseudo-*probabilities (i.e., the probabilities predicted by the model) of the peptides to belong to the active and inactive classes. These scores were subsequently weighted by the similarity to the training data to obtain similarity-weighted scores that consider the model’s applicability domain (ϕ_ACP_, ϕ_Neg,_ Eqs 5 and 6).

From each peptide library, we selected the two peptides with the highest ϕ_ACP_ and ϕ_Neg_ scores. None of the peptides were found in the training data or the CancerPPD database. No peptides were retrieved from the CancerPPD database with >95% similarity to the selected ones, as determined by the CD-HIT program^36^. We finally synthesized the 12 peptides and determined their half-effective concentration (EC_50)_ values against the MCF7 and A549 cancer cell lines. For 10 of the 12 synthesized peptides, the predictions were correct (Table 4). All of the peptides predicted to be inactive did not kill more than 50% of the cancer cells at a concentration of 50 µM. Of the six peptides predicted to be active, two were determined to be false positives (inactive at 50 µM) (Figs S10 and S11, Supplementary Information). Of the four correctly predicted active peptides, three were active in a low-micromolar range against both of the tested cancer cell lines, and the fourth (*Gradient2*) showed activity solely against MCF7 cells (Table 4).

**Table 4:** Experimental validation of the SVM prediction model.

Peptide	Sequence^a^	ϕ_ACP_	ϕ_Neg_	Prediction^b^	MCF7 EC_50_/µM	A549 EC_50_/µM	Outcome^c^
Helical1	FLWIKLGKLAGAVLKLILGLKKVV	0.94	0.45	+	4.4 ± 1.3	8.3 ± 2.0	TP
Helical2	GLWAIAVKAGKVILKLIVFIWIRV	0.94	0.45	+	>50	>50	FP
Helical3	GLLDIAGGNAETLAGHAV	0.44	0.90	−	>50	>50	TN
Helical4	GLFDVIGSQAGGAAPHFLG	0.46	0.89	−	>50	>50	TN
AmphiArc1	KWVKKVHNWLRRWIKVFEALFG	0.96	0.46	+	7.0 ± 0.5	18.4 ± 0.7	TP
AmphiArc2	KIFKKFKTIIKKVWRIFGRF	0.95	0.46	+	5.7 ± 0.7	9.3 ± 1.5	TP
AmphiArc3	AFRHSVKEELNYIRRRLERFPNRL	0.42	0.91	−	>50	>50	TN
AmphiArc4	RIENGLRKRLQSIYRHLEE	0.42	0.91	−	>50	>50	TN
Gradient1	KWVRIWIKVLRGLFVWVWFF	0.96	0.46	+	>50	>50	FP
Gradient2	AWLKRIKKFLKALFWVWVW	0.96	0.46	+	19.0 ± 1.8	>50	TP
Gradient3	KVVDNFENILII	0.40	0.85	−	>50	>50	TN
Gradient4	RVNAAIPNIIV	0.41	0.84	−	>50	>50	TN

The peptides from each virtually designed library were evaluated according to a similarity-weighted score for belonging to the positive (ϕ_ACP_) and negative (ϕ_*Neg*_) class. The two peptides with the highest ϕ_ACP_ and ϕ_Neg_ scores for each library were synthesized and tested for anticancer activity on breast adenocarcinoma (MCF7) and lung adenocarcinoma (A549) cell lines (EC_50_, *mean* ± *std*, *N* = 3).

^a^All peptides were synthesized with amidated C-termini; ^b^Prediction: +predicted to be active, − predicted to be inactive; ^c^Outcome: TP: true positive, FP: false positive, TN: true negative.

**Figure 1:**
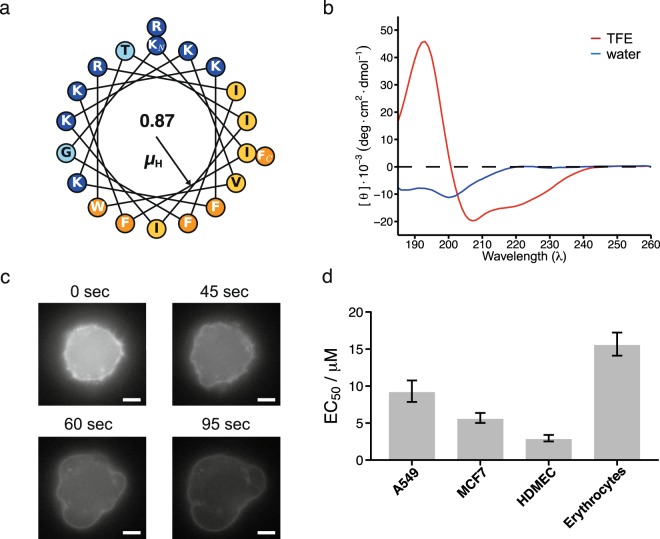
Characterization of the AmphiArc2 peptide. (**a**) Helical wheel plot of the peptide sequence with annotated hydrophobic moment direction and magnitude (*µ*_*H*_). Polar residues are shown in light blue, positively charged residues in dark blue, hydrophobic residues in yellow, and aromatic residues in orange. (**b**) Circular dichroism spectra of the peptide in water (blue) and in a 50% v/v TFE:water solution (red). (**c**) Time sequence of cell death of a single MCF7 cell trapped in a microfluidic chamber after exposure to the AmphiArc2 peptide. The cells were fluorescently labeled with calcein-AM dye in the cytosol, and their membrane was stained with fluorescently labeled EpCAM antibody. The scale bar represents 10 µm. (**d**) EC_50_ values of the peptide activity against the A549 and MCF7 cancer cells, noncancer HDMEC primary cells and the hemolytic activity (HC_50_) value of the peptide activity against human erythrocytes are shown. Error bars show the standard deviation of *N* = 3 independent experiments.

The AmphiArc2 peptide, the shortest peptide of the low micromolar active peptides, has a high hydrophobic moment (µ_H_ = 0.87) and a 180° arc of hydrophobic residues in an idealized helical structure (Fig. 1a). As determined by circular dichroism (CD) spectroscopy, the AmphiArc2 peptide is unstructured in pure water but adopts an alpha-helical structure in a hydrophobic environment (in 50% v/v water:2,2-trifluoroethanol, TFE) (Fig. 1b). Helix formation in a hydrophobic, membrane-like environment has been shown to be a characteristic of certain alpha-helical AMPs and ACPs^37,38^. To further investigate its membranolytic action, we observed the activity of AmphiArc2 on single MCF7 cells entrapped in a microfluidic chip. Video recordings showed morphological changes in the cell membrane and leakage of the cytosolic components as early as 30 seconds after initial contact with the peptide in the cells (Fig. 1c, Supplementary Information, Video SV1). After 95 seconds, the dye encapsulated in the cancer cell had leaked out, and the cell membrane showed deformations and blebbing.

After characterizing the anticancer activity of the AmphiArc2 peptide, we tested its cell-type selectivity. We determined its EC_50_ value against the noncancer HDMEC primary cell line and half-effective hemolytic concentration (HC_50_) against human erythrocytes (Fig. 1d). Both values were found to be in the same low-micromolar range as the EC_50_ against cancer cell lines, indicating toxicity of this peptide against noncancer cells.

### Selectivity optimization of a *de novo* designed ACP

**Figure 2:**
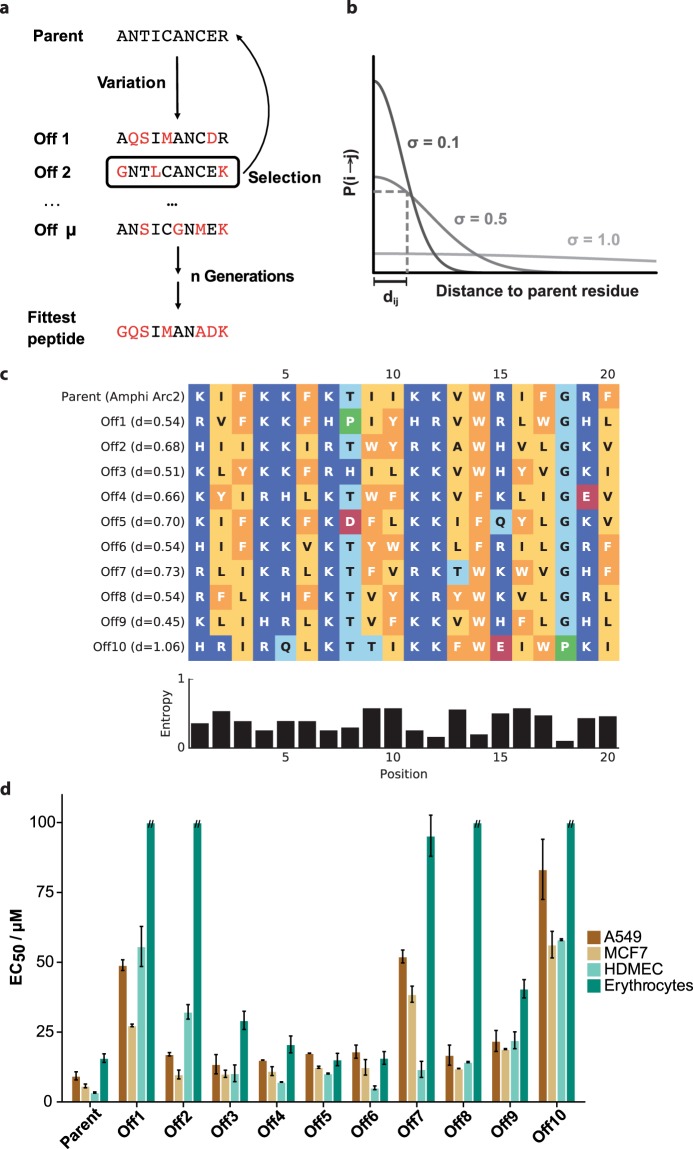
Peptide selectivity optimization by simulated molecular evolution (SME). (**a**) Principle of the iterative variation or mutation and selection steps in SME, starting with the model parent peptide “ANTICANCER”. (**b**) Probability of the mutation of amino acid residue *i* in the parent sequence to residue *j* in the offspring as a function of the amino acid pairwise similarity (*d*_*ij*_). The sigma (*σ*) parameter controls the sequence diversity among the offspring. (**c**) Comparison of the 10 generated offspring sequences and their Euclidean distance to the parent sequence according to the Grantham similarity matrix. The [0, 1] normalized Shannon entropy (in bit in the graph) of each residue position is shown below. Residue coloring is as follows: light blue: polar, dark blue: positively ionizable, red: negatively charged, yellow: hydrophobic, orange: aromatic, green: proline. (**d**) Peptide activity towards the A549 and MCF7 cancer cell lines (EC_50_), the noncancer HDMEC primary cells (EC_50_), and the human erythrocytes (HC_50_). The error bars give the standard deviation of *N* = 2 independent measurements with six technical replicates each.

We applied the SME algorithm to improve the selectivity of the AmphiArc2 peptide towards noncancer cells. SME contained a variation (mutation) and a selection operator (Fig. 2a). By variation, a series of offspring was generated from a parent sequence. The fittest offspring of a generation was selected and used as a parent in the next SME iteration. In this study, parents were selected among the offspring that maintained anticancer activity but showed enhanced selectivity for cancer cells (selection operator). The mutations in the sequence variation step were performed according to a normalized Gaussian probability distribution of pairwise amino acid similarity (*d*_*ij*_) (Fig. 2b). As a similarity measure, we utilized the Grantham matrix, which takes into account the atom composition, the polarity and the molecular volume of the residues^39^. The probability of substitution of residue *i* to residue *j* decreases with decreasing pairwise amino acid similarity. The degree of similarity of the offspring peptides to the parent sequence (offspring diversity) was controlled via the sigma (*σ*) parameter (Fig. 2b). A higher sigma value allowed the generation of sequences further away from the parent peptide (Fig. S5, Supplementary Information).

We performed a total of three SME iterations, starting from the AmphiArc2 peptide. In the first iteration, we generated 10 offspring peptides with *σ* = 0.1 (Fig. 2c). The mutations introduced by this sigma value were conservative amino acid changes that maintained the overall amphipathicity of the peptide. We synthesized and tested all ten offspring peptides of the three SME generations against the MCF7 and A549 cancer cell lines to determine their anticancer activity. For selectivity assessment, we tested their activity against the noncancer HDMEC primary cell line and measured their hemolytic activity on human erythrocytes (Fig. 2d).

**Figure 3:**
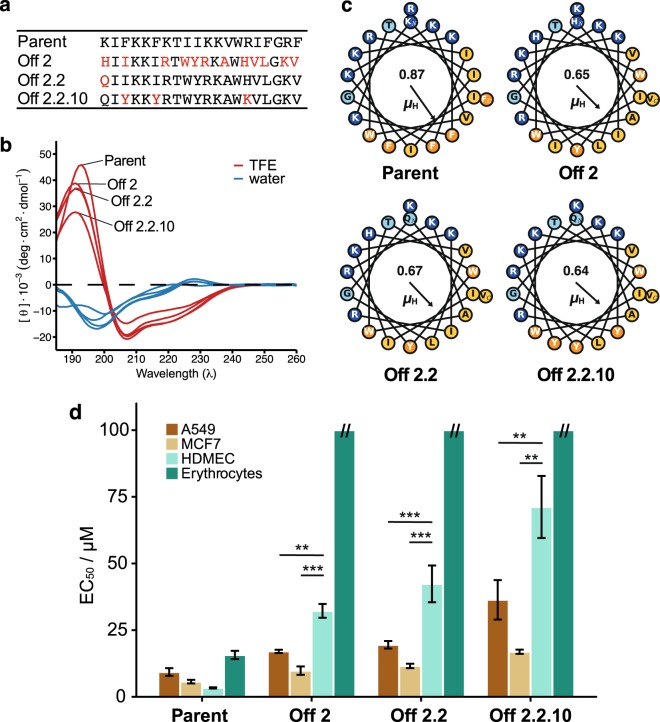
Characterization of the parent peptides and the most selective offspring peptides from three subsequent SME generations. (**a**) Amino acid sequences; red residues denote sequence changes from the respective parent sequence. (**b**) Circular dichroism spectra in water (blue) and a mixture of 50% v/v TFE:water. (**c**) Helical wheel plots with hydrophobic moment direction and magnitude (µ_H_). Residue coloring: polar residues in light blue, positively ionizable residues in dark blue, hydrophobic residues in yellow, and aromatic residues in orange. (**d**) Peptide activity towards the A549 and MCF7 cancer cell lines (EC_50_), the noncancer HDMEC primary cells (EC_50_), and the human erythrocytes (HC_50_). The error bars represent the standard deviation of three independent measurements. **p-value < 0.01, ***p-value < 0.001 of the mean differences (Welch t-test).

The results obtained demonstrate that small conservative amino acid replacements affected the activity and selectivity of these ACPs while conserving their overall amphipathic helical structure in a lipophilic environment. Offspring n.2 (Off2) maintained the low-micromolar activity of the AmphiArc2 peptide against the A549 and MCF7 cancer cells but showed a 12-fold reduction of hemolytic activity against human erythrocytes and a ten-fold reduction of activity against HDMEC cells (Fig. 2d). Therefore, we selected Off2 as the parent for the next SME iteration (Fig. 3), in which ten new peptides were generated (Off2.1 to Off2.10).

The second generation of peptide variation did not achieve meaningful selectivity improvements with respect to HDMEC cells (Fig. S7, Supplementary Information). Five of the offspring peptides (Off2.1, Off2.3, Off2.4, Off2.9, Off2.10) were inactive. This loss of activity correlated with the introduction of a proline residue in the sequence (Fig. S7, Supplementary Information). Prolines affect alpha-helical conformation by introducing helix kinks and breaks^40^. We corroborated this secondary structure disruption with circular dichroism analysis of Off2.1, Off2.3, Off2.4 and Off2.9 (Fig. S9, Supplementary Information).

In the third SME generation (Off2.2.1 – Off2.2.10), we actively omitted proline residues and reduced the sigma value from 0.1 to 0.06 to explore close analogs of Off2 and Off2.2 (Fig. S8, Supplementary Information). Off2.2.10 showed decreased activity towards the noncancer HDMEC primary cells (Fig. 3d). This increase in selectivity was accompanied by a decreased activity against both the A549 and MCF7 cell lines.

The most active, but nonselective, AmphiArc2 parent peptide and the most cancer-cell selective Off2.2.10 peptide possess several differences and commonalities in their physicochemical properties. Even though both peptides display a hydrophobic arc of 180°, the hydrophobic moment of Off2.2.10 (µ_H_ = 0.64) is lower than that of AmphiArc2 (µ_H_ = 0.87) (Fig. 3c). The parent peptide bears eight positive charges, while Off2.2.10 contains seven positively ionizable residues caused by the N-terminal K1Q mutation. This moderate reduction of both the hydrophobic moment and the net positive charge improved the peptide selectivity for cancer cells and reduced the risk of killing non-transformed cells. To further explore these sequence features, we analyzed the ratios of the EC_50_ in the noncancer cells and in the cancer cell lines of all tested peptides. The more selective peptides (higher EC_50_ ratio) are characterized by moderate hydrophobic moments and charge densities (Supplementary Information, Fig. S10), suggesting a guideline for optimizing the cancer-cell selectivity of ACPs. This observation is in accordance with reports stating that decreasing the hydrophobic moment of helical ACPs reduces both their hemolytic potential and anticancer activity^7–9^.

### NCI-60 cancer cell panel testing

The ACP candidates AmphiArc2 (parent), Off2 and Off2.2.10 were tested on the NCI-60 cancer cell panel^41^. The three tested peptides inhibited the growth of all the cancer cell lines in the NCI-60 panel at a low micromolar concentration (Table 5, Supplementary Information Table S3). This result corroborated the wide-spectrum effect of the anticancer peptides across a range of cancer types. Both the activity of Off2 and Off2.2.10 peptides on the cell lines tested were significantly lower than the anticancer activity of the AmphiArc2 peptide (*p*-value = 4.9 × 10^−13^ and 1.7 × 10^−12^, respectively, Welch two sample t-test), suggesting that the initial increased cancer cell selectivity comes at a cost of an activity loss. No significant anticancer activity difference was found between Off2 and Off2.2.10 peptides (*p*-value = 0.66, Welch two sample t-test), indicating that the additionally improved anticancer selectivity does not affect the average anticancer activities of these two peptides.

**Table 5:** Cellular growth inhibition of 60 cell lines in the NCI-60 cancer cell test for the AmphiArc2 (Parent), Off2 and Off2.2.10 peptides.

	AmphiArc2 log GI_50_	Off2 log GI_50_	Off2.2.10 log GI_50_
Leukemia	−5.5	−5.2	−5.3
Lung	−5.6	−5.4	−5.2
Colon	−5.6	−5.2	−5.1
CNS	−5.6	−5.2	−5.2
Melanoma	−5.6	−5.3	−5.2
Ovarian	−5.6	−5.2	−5.2
Renal	−5.7	−5.3	−5.2
Prostate	−5.7	−5.5	−5.4
Breast	−5.6	−5.4	−5.4

The averaged peptide activity for the cancer types tested is shown as the logarithm of the half growth inhibitory concentration (GI_50_, Supplementary Information Eq. S1), which is the molar concentration of peptide needed to inhibit half of the normal cancer cell growth. The logarithm of GI_50_ is shown here as 10^n^ M. The values from −5 to −6 correspond to growth inhibition in the 1–10 µM range. The growth inhibition values for the individual cell lines are displayed in Supplementary Information Table S4.

## Conclusions

In this study, the combination of a machine learning model and the SME algorithm resulted in ACPs with low-micromolar potency against a wide variety of cancer cells (NCI-60 panel) and selectivity with respect to non-transformed cells (HDMEC) and human erythrocytes. The machine-learning classifier alone was able to identify active peptides but was insufficient to identify cancer cell selective peptides. Virtual screening of computationally designed peptide libraries with the implemented machine-learning classifier led to the discovery of four novel ACPs as the starting point for selectivity optimization by SME. In the first design-synthesize-test cycle, peptide hemolysis was reduced ten-fold, and after three cycles, peptide activity towards noncancer cells was reduced more than 20-fold while retaining anticancer activity compared to the parent peptide (AmphiArc2). The results of this study advocate for the SME method for experiment-guided peptide design and for exploration of the ACP structure-activity landscape. SME is applicable to all kinds of experimental readouts and provides an alternative to more conventional peptide optimization techniques, *e.g*., alanine scanning. At the same time, the results suggest that additionally increased cancer cell selectivity of membranolytic ACPs might come at the price of reduced peptide potency. This working hypothesis provides a basis for future study.

## Methods

### Machine learning model

Both machine learning models were constructed in Python v2.7 using the Scikit-Learn v0.18 library. For model training, the peptide dataset was split into 2/3 training and 1/3 testing subsets. Random forest classifier: the number of trees (“n_estimators”) was set to 500, and the number of features to be considered by each tree (“max_features”) was set to the squared root of all features (“sqrt”). SVM classifier: a linear kernel was employed and hyperparameter *C* was optimized by a ten-fold cross-validation in which the model is trained on 90% of the training data and validated on the remaining 10% in ten repetitions of training. The obtained mean of the 10 repetitions (cross-validation MCC score) was used to evaluate the performance of the models. The test scores were obtained with the independent test set.

#### Scoring metrics

The Matthews correlation coefficient (MCC, Eq. 1), accuracy (Eq. 2), precision (Eq. 3) and recall (Eq. 4) were calculated. *TP, FP, TN* and *FN* correspond to the number of true positives, false positives, true negatives and false negatives predicted by the model, respectively.

1 $$MCC=\frac{TP\times TN-FP\times FN}{\sqrt{(TP+FP)(TP+FN)(TN+FP)(TN+FN)}}$$

2 $$Accuracy=\frac{TN+TP}{TN+TP+FN+FP}$$

3 $$Precision=\frac{TP}{TP+FP}$$

4 $$Recall=\frac{TP}{TP+FN}$$

#### Data weighted scoring functions

To appropriately consider the applicability domain of the SVM classifier, the final scoring function for ACPs (ϕ_ACP_, Eq. 5) and inactive (negative) peptides (ϕ_Neg_, Eq. 6) considers both the *pseudo-probability* of the peptide to be an ACP (*P*_*ACP*)_ as predicted by the SVM model and the similarity of the predicted peptides to the training data (*Sim. score*). *k*-means clustering with *k* = 3 was performed with Python v2.7 and the Scikit-Learn v0.18 library package. The similarity score is calculated as the inverse of the Euclidean distance in descriptor space of the peptides to the three centroids.

5 $${\varphi }_{ACP}=\frac{{P}_{ACP}+Sim.\,score}{2}$$

6 $${\varphi }_{Neg}=\frac{(1-{P}_{ACP})+Sim.\,score}{2}$$

### Virtual peptide libraries

Three virtual peptide libraries were generated according to three different design principles. For each library, the peptide length was restricted to a range of 11 to 30 amino acids, as peptides able to fold in an alpha-helix are typically inside this range^42^. Duplicate sequences were eliminated, and the similarity of the sequences was restricted with the *CD-HIT*^36^ program to a threshold of 0.8 similarity. A total of 10^6^ peptides were selected from each of the libraries.

- *Helical library*. The Helical library was generated with the position-dependent amino acid distributions of 62 anuran and hymenopteran alpha-helical ACPs^11^ in amino acid positions 1–18 (exactly 5 helical turns). For longer peptides, the pattern was repeated. The method to generate this library is included in the modlAMP^43^ Python package (*modlamp.sequences.HelicesACP*).
- *Amphipathic Arc library*. The design principle of the Amphipathic Arc library was amphipathic peptide sequences, which would potentially be alpha-helical with a preference for positively charged amino acids in the polar phase of the helix and varying hydrophobic arcs in the range 100–260°. The method to generate this library was included in the python package *modlAMP* as the class AmphipathicArc (*modlamp.sequences.AmphipathicArc*).
- *Gradient library*. The Gradient library was designed using the same procedure as the Amphipathic Arc library but with an additional hydrophobic gradient in the peptide structure from the N- to the C-terminus. For this, the amino acids in the C-terminal third of the peptide sequence were substituted with hydrophobic amino acids. In the *modlAMP* package, this was achieved by the method *make_H_gradient* in the *modlamp.sequences.Amphipathic Arc* class.

### Simulated molecular evolution

The simulated molecular evolution (SME) algorithm is based on the (1, λ) evolution strategy^44^ in which λ mutated sequences (offspring) are generated from a parent sequence^22,23,25^. The offspring was scored according to a fitness function, which was defined as the experimentally determined peptide anticancer activity and selectivity with respect to non-transformed cells. The best offspring were selected as a parent for the following optimization iteration. The amino acid mutations were generated according to an amino acid similarity matrix that has been row-normalized (*d*_*ij*_) to allow for a *pseudo*-probability calculation of the amino acid transitions (Eq. 7). Here, the Grantham amino-acid similarity matrix was utilized^39^. The amino acids cysteine and methionine were excluded from the mutation matrix to avoid potential peptide cyclization and facilitate peptide synthesis.

7 $$P\,(i\to j)=exp(-\frac{{d}_{ij}^{2}}{2{\sigma }^{2}})/\sum _{j}\,exp(-\frac{{d}_{ij}^{2}}{2{\sigma }^{2}}).$$

where *σ* is a strategy parameter that controls the distance of the offspring sequences to the parent sequence and, thus, the sequence diversity among the offspring. The *σ* strategy parameter was set to 0.1 for the two initial SME iterations. Sequence diversity was characterized by the Shannon entropy^45^ (H) of the residue distribution among the offspring (Eq. 8), where *p*_i_ corresponds to the frequency of amino acid *i* in a certain sequence position. The Shannon entropy values were normalized to [0, 1]. The simulated molecular evolution strategy and Shannon entropy calculation were programmed with Python v2.7.

8 $$H=\sum _{i=1}^{20}\,{p}_{i}lo{g}_{2}{p}_{i}.$$

## Supplementary information

Video SV1

Supplementary Information

Supplementary Dataset 1
