# Supplementary material for: In silico design and optimization of selective membranolytic anticancer peptides
Source: Sci Rep. 2019 Aug 2;9:11282. doi: 10.1038/s41598-019-47568-9 (PMC6677754; doi:10.1038/s41598-019-47568-9)
Supplement: Supplementary file 2 — Supplementary Information [file 41598_2019_47568_MOESM2_ESM.pdf]

Supporting information

In silico design and optimization of selective membranolytic anticancer peptides

Gisela Gabernet[1], Damian Gautschi[1], Alex T. Müller[1], Claudia S. Neuhaus[1], Lucas Armbrecht[2], Petra S. Dittrich[2], Jan A. Hiss[1], and Gisbert Schneider[1]*
[1]Department of Chemistry and Applied Biosciences, ETH Zurich, Zurich, Switzerland
[2]Department of Biosystems Science and Engineering, Swiss Federal Institute of Technology (ETH), Basel, Switzerland
* E-Mail: gisbert.schneider@pharma.ethz.ch

Supplementary figures and tables 2
Feature selection 2
Model performance comparison 3
Feature importance analysis 4
Characterization of virtual peptide libraries 5
Distribution of ACP scores for the virtual peptide libraries 6
Influence of the strategy parameter on the offspring diversity 7
Peptide sequences and cell activity values for three simulated molecular evolution generations 8
Circular dichroism measurements 11
Data set construction and feature calculation 13
Data set construction 13
Feature calculation 13
Feature selection 14
Experimental methods 15
Peptide synthesis 15
Peptide purification 15
Circular dichroism 15
Cancer cell cultures 15
Cell viability assay 16
Hemolysis assay 16
Microfluidics chip experiments 17
NCI-60 screening procedure 17
In vitro dose-response data 18
Dose-response data for peptides in Table 1 18
Dose-response data for the SME offspring 20
Dose-response data on the NCI-60 cell panel 21
Supplementary files 23
Supplementary Video SV1 23
Training data set 23
References 23

Supplementary figures and tables

Feature selection

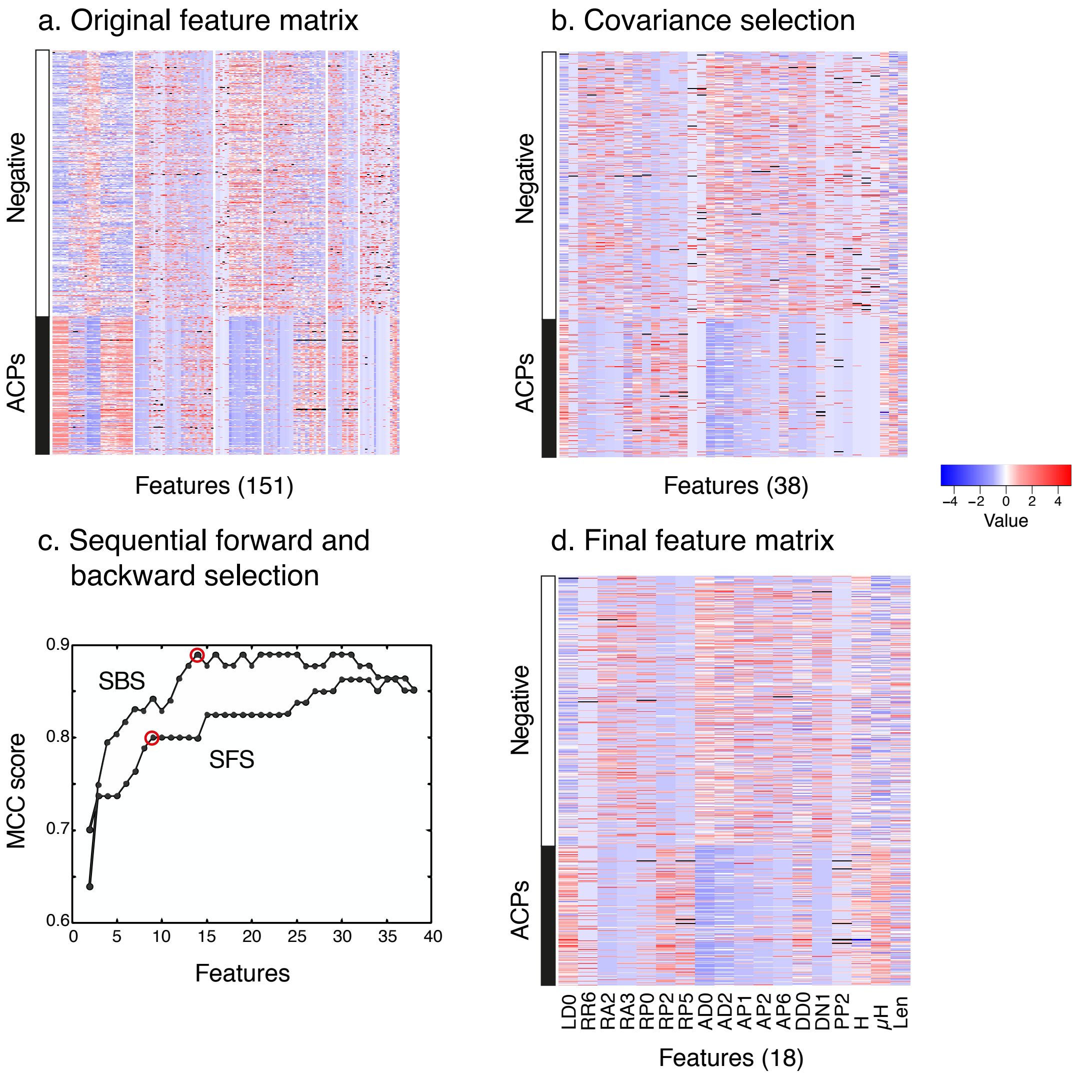

Figure S1. Feature selection steps. **a**. Original feature matrix. **b**. Matrix after covariance selection feature elimination. **c**. Matthews correlation coefficient (MCC) score values during the sequential forward and backward elimination steps. Selected number of features in each of the iterations are circled in red. **d**. Feature matrix of the after all the selection steps.

Model performance comparison

Table S1. Online available ACP prediction tools. All tools are based on an SVM classifier model but employ different training data sources and peptide descriptors.

Model	Positive training data	Negative training data	Descriptor(s)
AntiCP model 1[1]	ACPs in CAMP[2] and DADP[3] databases	AMPs in APD[4] database	Freq. of amino acids.<br>Amino acid pairs.<br>Ordered amino acid composition of 5-20 residues in the N and C-terminal positions.
AntiCP model 2[1]	ACPs in CAMP and DADP databases	Random peptides from SwissProt[5] database	Freq. of amino acids.<br>Amino acid pairs.<br>Ordered amino acid composition of 5-20 residues in the N and C-terminal positions.
iACP[6]	ACPs in APD database	Non-secretory proteins from Uniprot[5] database	Composition of amino acid pairs separated by a gap of 0 to 4 residues, and a feature selection procedure.
MLACP[7]	ACPs in CAMP and DADP databases	Random peptides from SwissProt database	Composition of amino acids and amino acid pairs, atomic composition and amino acid physicochemical properties.

Feature importance analysis

The surface normal vector to the hyperplane of an SVM with a linear kernel can be analytically described as a linear combination of the input features. The magnitude of the weights attributed to each feature corresponds to their importance for the classification problem, and their positive or negative sign indicates whether the feature is found in the positive or the negative data class, respectively. Positive weights were assigned by the model for global hydrophobicity (H), hydrophobic moment (μ_H) and positive charge (PPd2). The peptide length was also positively weighted by the model. The two features with the greatest weight values take into account the frequency of amino acids with hydrogen-bond donor and acceptor groups (ADd0, DDd0).

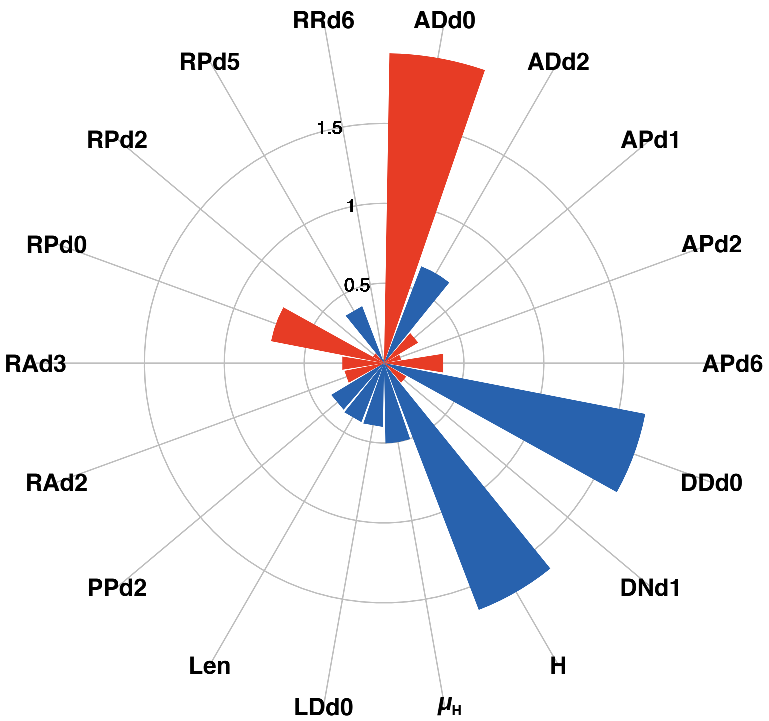

Figure S2. Feature importance of the 18 features obtained after covariance elimination and sequential feature selection. The weight magnitudes and signs of the applied to the feature matrix as obtained from the SVM classifier model are plotted. Red: weight attributed to the negative class, blue: weight attributed to the positive class (here: ACP). The abbreviations are explained in Table S2.

Characterization of virtual peptide libraries

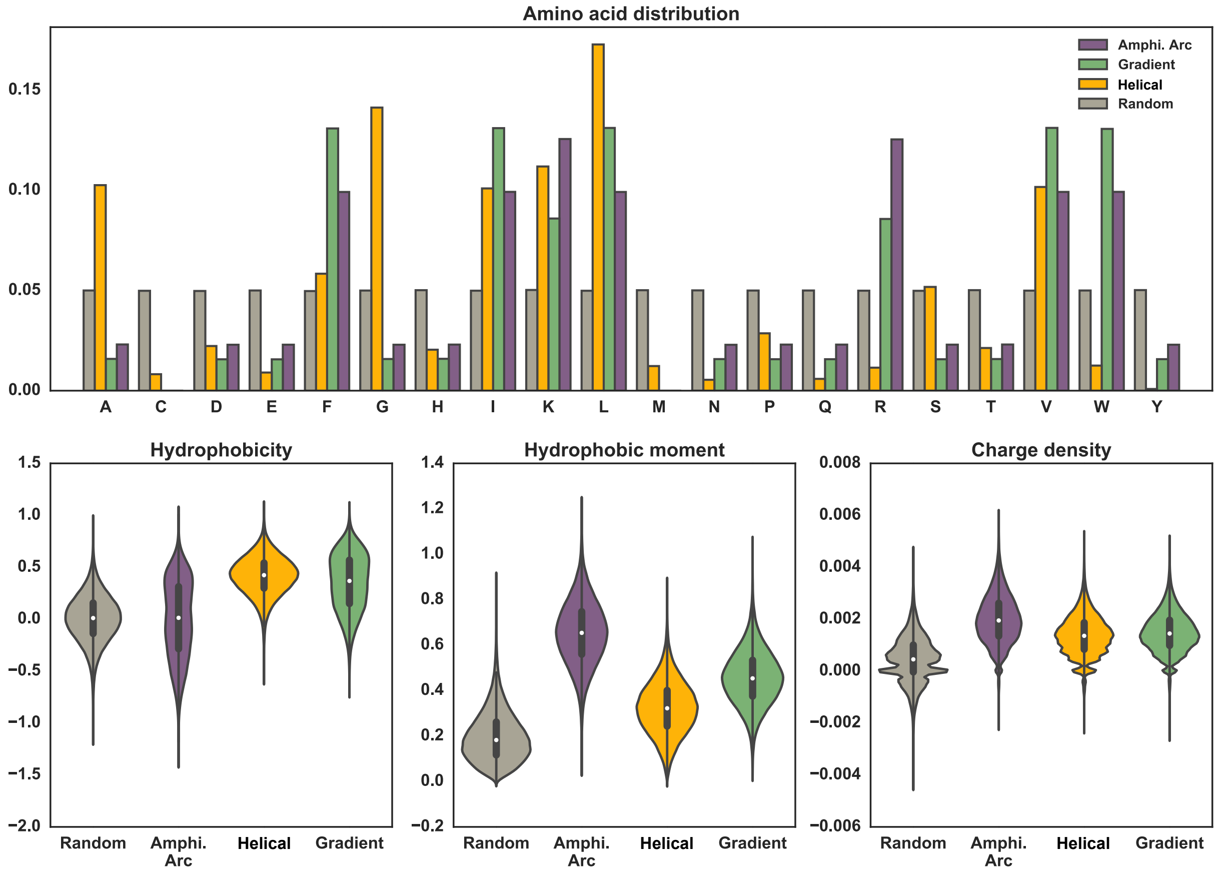

Figure S3. Amino acid distribution and physicochemical properties of the three generated virtual peptide libraries (Amphipathic Arc, Helical and Gradient) in comparison with a peptide library with a random amino acid distribution. Hydrophobicity and hydrophobic moment were calculated with the Eisenberg hydrophobicity scale and the charge density at pH 7 are shown. Descriptor calculations were performed with the *modlAMP* package[9].

Distribution of ACP scores for the virtual peptide libraries

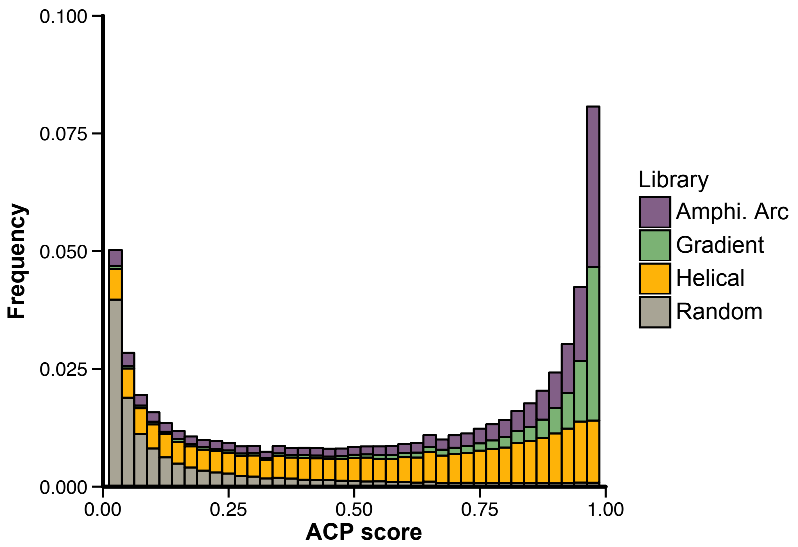

Figure S4. Distribution of the data-weighted scores (σ_{ACP}) for the three generated virtual peptide libraries (Amphipathic Arc, Helical and Gradient) in comparison with the scores for a peptide library generated with a random amino acid distribution (Random).

Influence of the strategy parameter on the offspring diversity

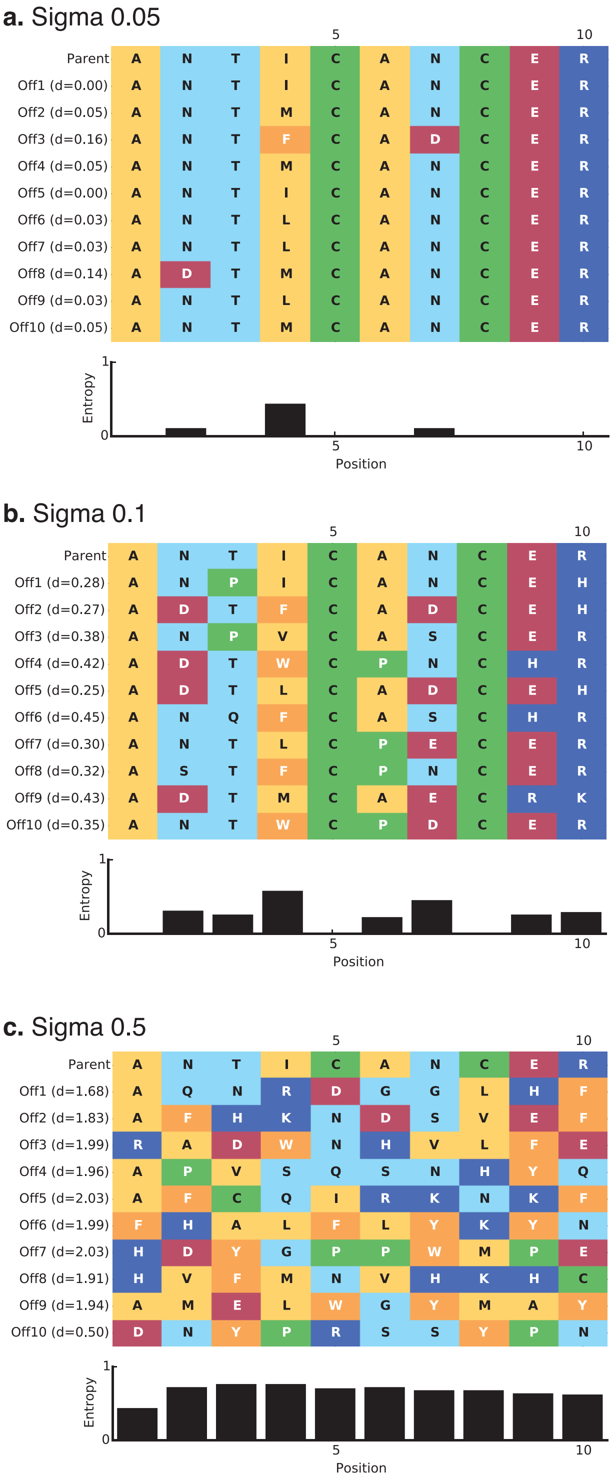

Figure S5. Influence of sigma in the diversity of the offspring sequences. Three simulated molecular evolution iterations were performed from the same parent peptide "ANTICANCER", using three different values for the strategy parameter (σ) (**a** = 0.05, **b** = 0.1, **c** =0.5). For higher sigma values, the position Shannon entropy increases, indicating higher offspring diversity.

Peptide sequences and cell activity values for three simulated molecular evolution generations

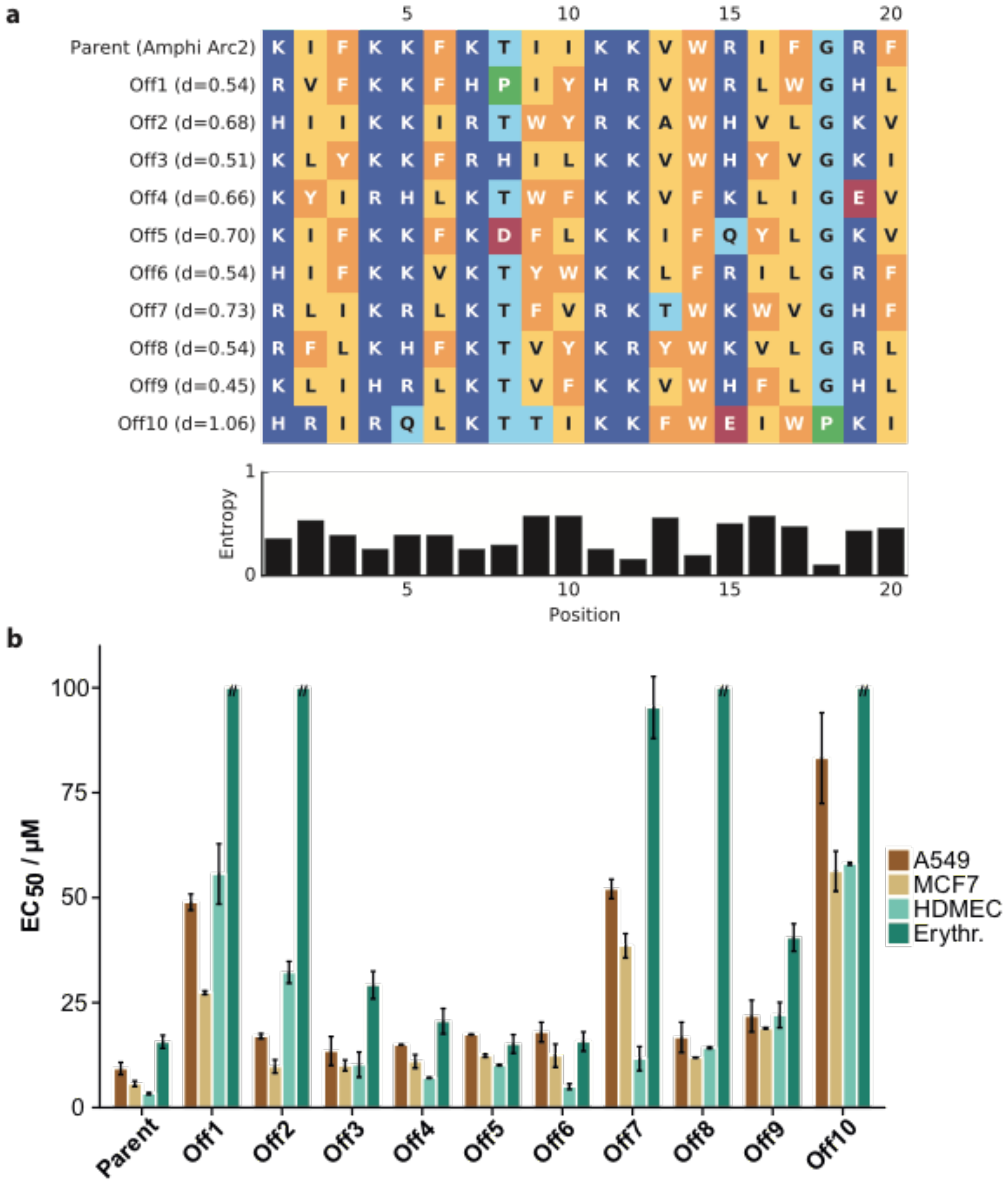

Figure S6. First iteration of simulated molecular evolution on the parent peptide AmphiArc2. **a**. Comparison of the ten generated offspring sequences (σ = 0.1) and their Euclidean distance to the parent sequence according to the Grantham similarity matrix. The Shannon entropy variation (in bit) of each residue position is shown below. Color coding for the amino acids is the following: hydrophobic (yellow), aromatic (orange), polar (blue), positively charged (dark blue), negatively charged (red). **b**. Anticancer activity of the peptides on the A549 and MCF7 cancer cell lines, and the HDMEC non-cancer primary cell line (EC_{50}). Hemolytic activity on human erythrocytes (HC_{50}). The error bars represent the standard deviation of N = 2 independent measurements with six technical replicates each for the anticancer activity determination and three technical replicates for hemolysis determination.

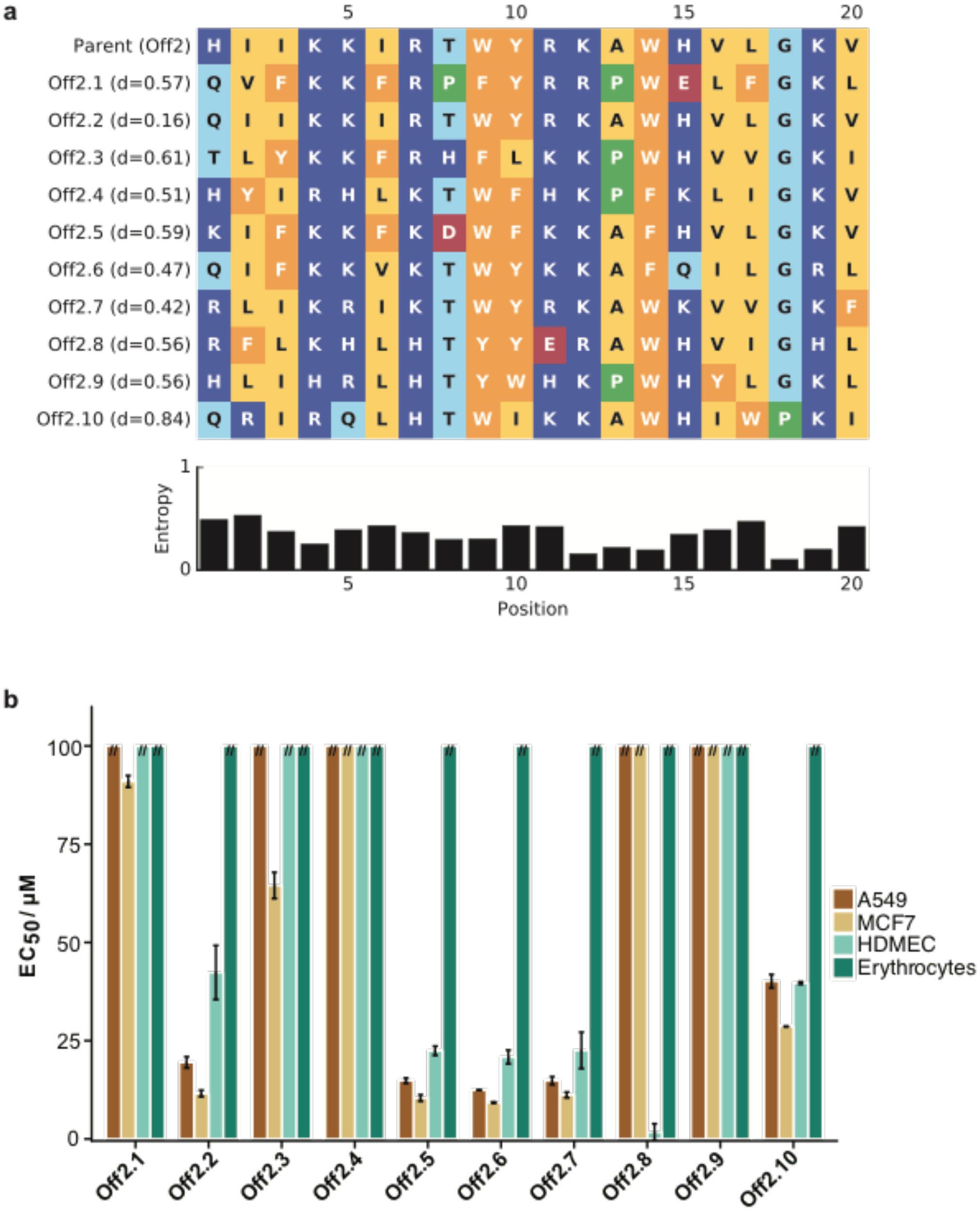

Figure S7. Second iteration of simulated molecular evolution on the parent peptide Off2. **a**. Comparison of the ten generated offspring sequences (σ = 0.1) and their Euclidean distance to the parent sequence according to the Grantham similarity matrix. The [0,1] normalized Shannon entropy (bit) of each residue position is shown below. Color coding for the amino acids is the following: hydrophobic (yellow), aromatic (orange), polar (blue), positively charged (dark blue), negatively charged (red). **b**. Anticancer activity of the peptides on the A549 and MCF-7 cancer cell lines, and the HDMEC non-cancer primary cell line (EC_{50}). Hemolytic activity on human erythrocytes (HC_{50}). The error bars represent the standard deviation of N = 2 independent measurements with six technical replicates each for the anticancer activity determination and three technical replicates for hemolysis determination.

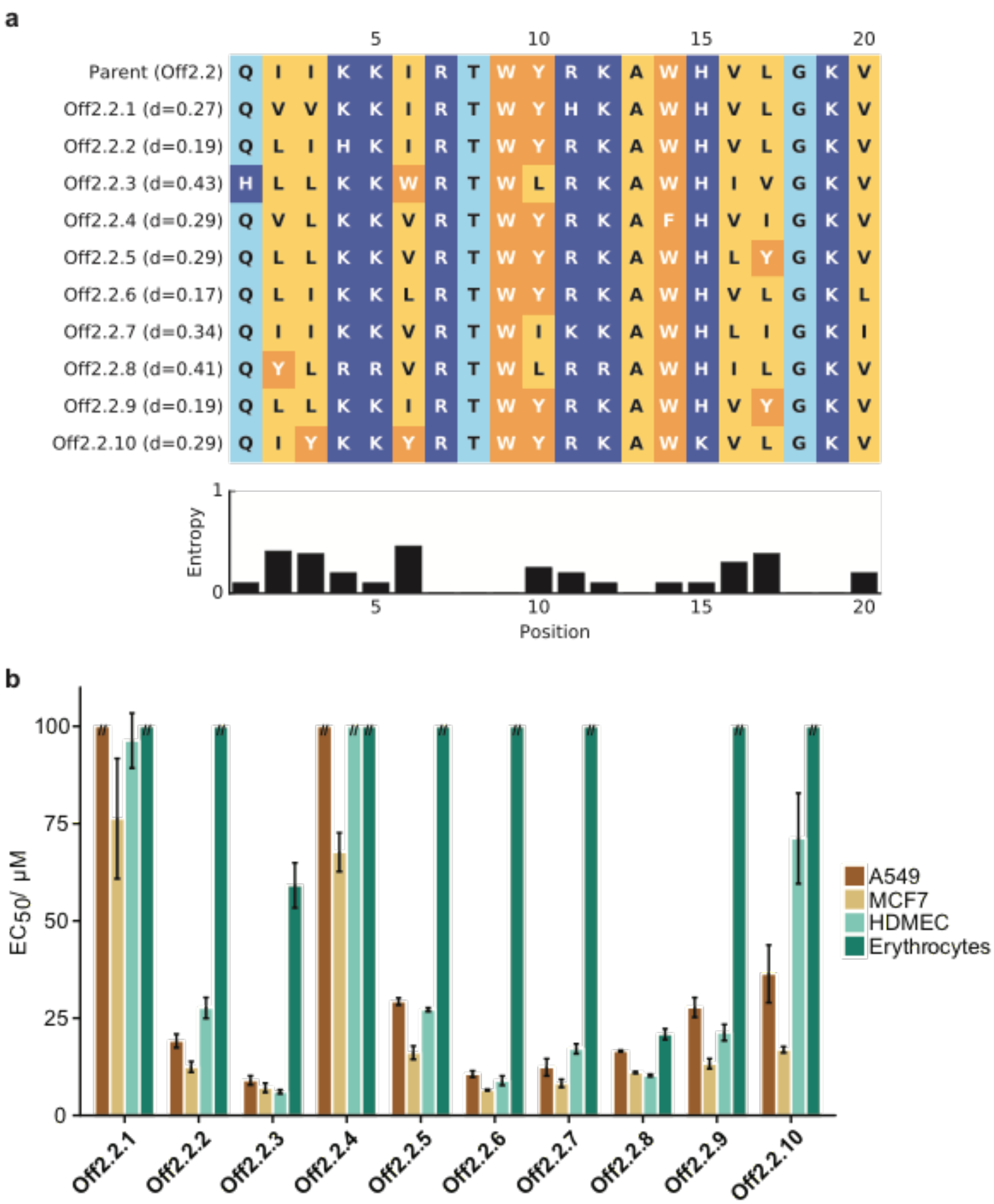

Figure S8. Third iteration of simulated molecular evolution on the parent peptide Off2.2. **a**. Comparison of the ten generated offspring sequences (σ = 0.06) and their Euclidean distance to the parent sequence according to the Grantham similarity matrix. The [0,1] normalized Shannon entropy (bit) of each residue position is shown below. Color coding for the amino acids is the following: hydrophobic (yellow), aromatic (orange), polar (blue), positively charged (dark blue), negatively charged (red). Proline residues were additionally removed from the mutation matrix to avoid secondary structure disruption. **b**. Anticancer activity of the peptides on the A549 and MCF-7 cancer cell lines, and the HDMEC non-cancer primary cell line (EC_{50}). Hemolytic activity on human erythrocytes (HC_{50}). The error bars represent the standard deviation of N = 2 independent measurements with six technical replicates each for the anticancer activity determination and three technical replicates for hemolysis determination.

Circular dichroism measurements

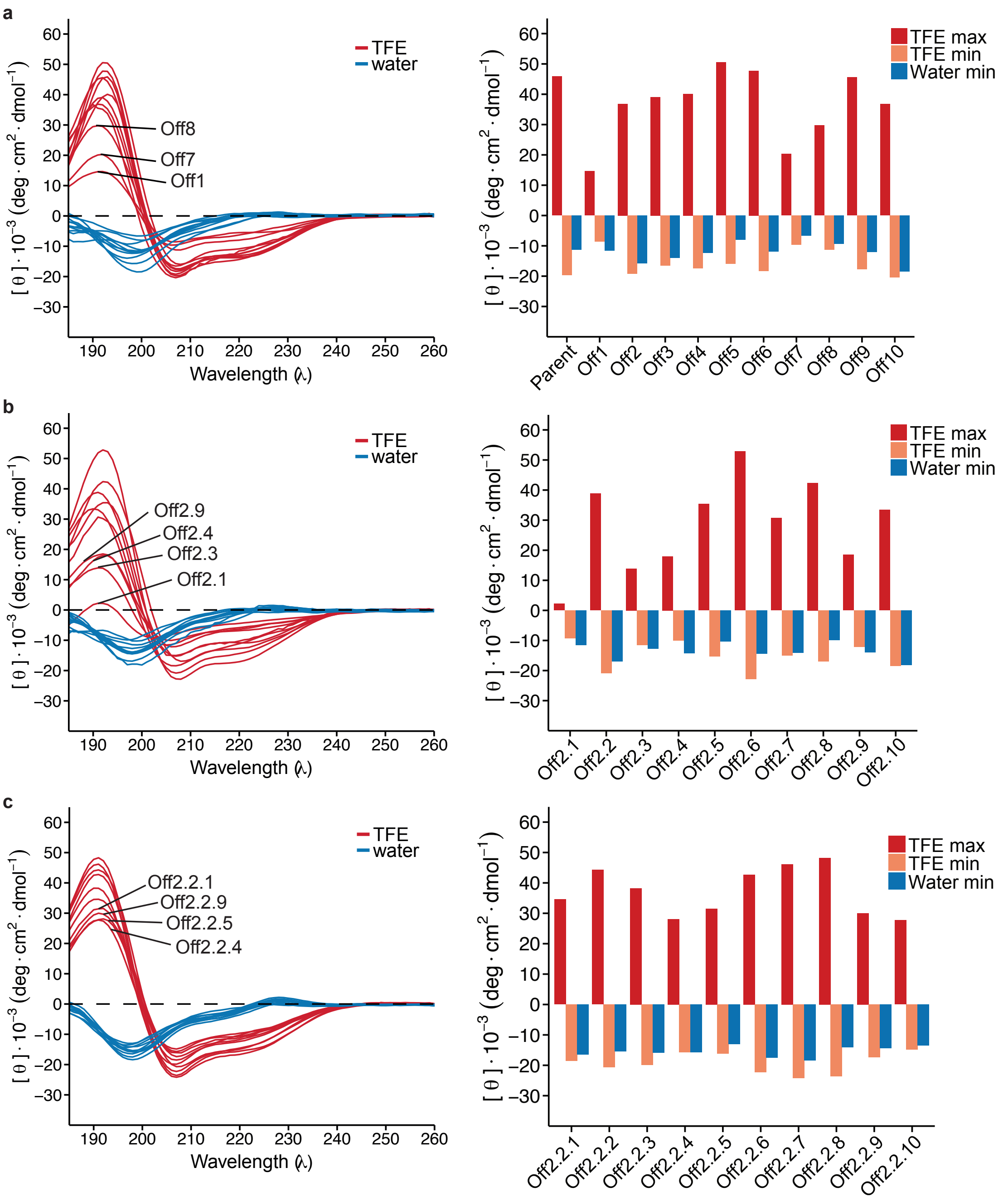

Figure S9. Circular dichroism recording for the offspring peptides in the three simulated molecular evolution generations. The circular dichroism spectra are shown for measurements in water (blue) and a 50% v/v water:2,2-trifluoroethanol (TFE) solution (red) for the peptides of the first (**a**), second (**b**), and third (**c**) iterations. The bar plots show the intensity of the maxima and minima for each of the peptides.

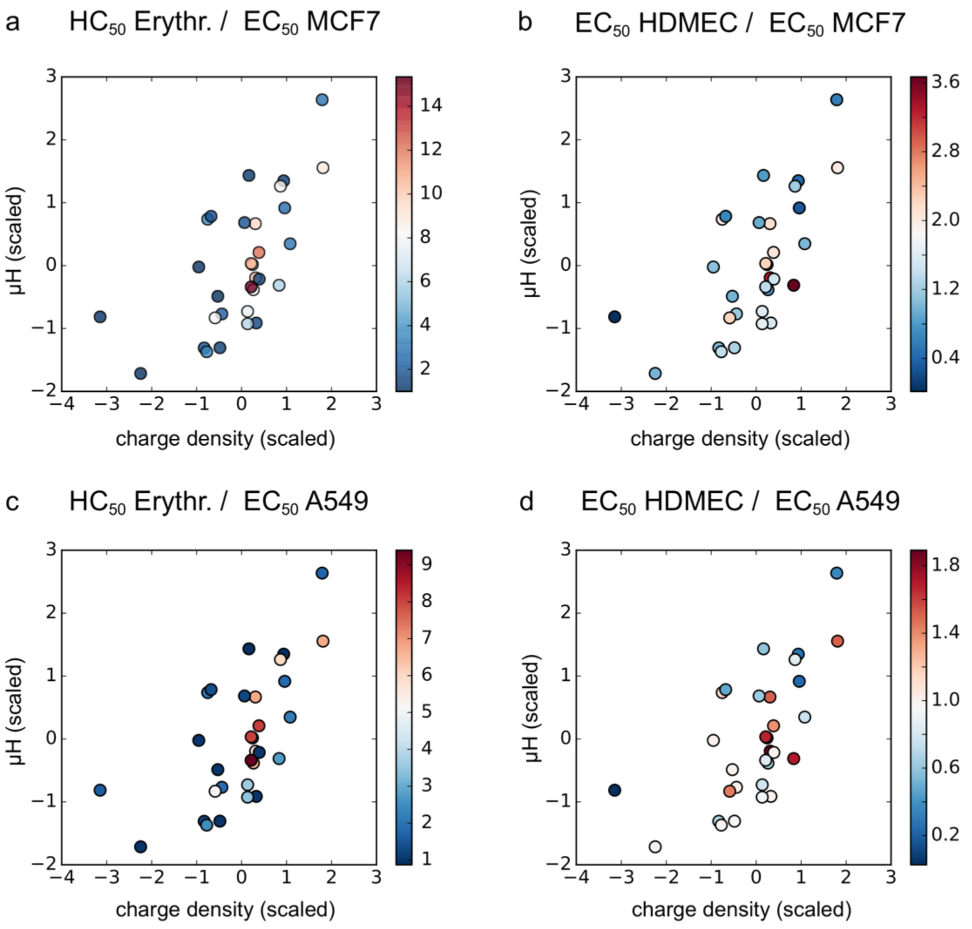

Figure S10. Dependence of ACP selectivity on the peptide hydrophobic moment (μ_H) and charge density. The ratio of anticancer activity (EC50) on the MCF7 and A549 cell lines with respect to hemolysis of human erythrocytes (HC50) (**a** and **c**), or HDMEC primary non-cancer cells (**b** and **d**) is represented by a color gradient. The peptides with higher ratios (red), and therefore more selective for cancer cells, cluster together in a region of moderate hydrophobic moment and charge density.

Data set construction and feature calculation

Data set construction

- *Positive dataset.* ACP sequences were retrieved from the CancerPPD[10] database (database accessed 02/06/2016) and duplicates were eliminated, obtaining a total of 539 peptides. Only linear, cysteine-free sequences were kept avoiding potential cyclization and dimerization in vitro. This set was further filtered to restrict the peptide length to 7-30 amino acids and to eliminate peptides containing non-canonical amino acids. The final set consisted of 339 ACPs.
- *Negative dataset.* Alpha-helical sequences from non-transmembrane proteins contained in the Protein Data Bank (PDB)[11] were used as the negative dataset for machine learning. The PDB IDs from "all-alpha" proteins representatives at 70% pairwise similarity were downloaded (PDB accessed on 10/12/2015), excluding transmembrane proteins. The individual alpha helices from these proteins were extracted with an R script[12], using the R packages *bio3d*[13] and *seqinr*[14]. The representative helices with a similarity threshold of 0.8 in *cd-hit*[15] were kept. The set was filtered in the same way as the positive data set to restrict the peptide length from seven to 30 amino acids and to eliminate peptides containing non-canonical amino acids and cysteine residues. From the resulting set of 12,277 peptides, 680 samples were randomly selected to obtain a data set with double the number of negative peptides than positive peptides. 25 in-house peptides that had previously been synthesized and tested inactive against cancer cells in our laboratory were included in the negative data set, resulting in a total of 705 negative, i.e. inactive peptides.

Feature calculation

The peptide descriptor matrix was calculated in Python v2.7 (www.python.org) by the use of the package *modlAMP*[9]. The following descriptors were included to obtain a 151-dimensional vector of molecular descriptors.

- *pepCATS descriptor*. The pepCATS descriptor[16] is based on a binary vector representing each canonical amino acid in terms of lipophilic (L), aromatic (R), hydrogen-bond acceptor (A), hydrogen-bond donor (D), positively (P) and negatively (N) ionizable states at physiological pH. These features are convoluted by the use of the Moreau-Broto cross-correlation[17] over a sliding window of seven amino acids to achieve a length-independent peptide descriptor. For the six pharmacophore features and a window of seven amino acids, the descriptor vector contains 147 elements.
- *Hydrophobicity.* The peptide hydrophobicity was calculated as the sum of the hydrophobicity values of the individual amino acids as given by the Eisenberg hydrophobicity scale[8].
- *Hydrophobic moment.* The largest hydrophobic moment for any window of 11 amino acids of the peptide was calculated as previously described by Eisenberg[8].
- *Charge density.* The peptide charge density was calculated as the net charge at pH 7 divided by the molecular weight.
- *Length.* The number of amino acid residues in a peptide.

Feature selection

A series of feature selection steps were sequentially applied to the descriptor matrix to reduce its feature redundancy and dimensionality, as described below.

- *Variance threshold.* Features with zero variance in the training set were eliminated using the "*VarianceThreshold*" class from *scikit-learn*[18]. This feature selection step reduced the dimensionality from 151 to 146 dimensions.
- *Covariance elimination.* Features with a covariance > 0.5 were identified, and for each group one feature was retained that contributed the most to the variance of the first principal component (PC1) of a principal component analysis (PCA)[19]. The covariance elimination method was programmed in Python v2.7, employing the class "*decomposition.PCA*" for the covariance matrix calculation and to assess the variance contribution of each features to PC1. This selection step reduced the feature matrix dimensionality to 38.
- *Sequential forward and backward feature elimination***.** In sequential forward selection, starting from an empty feature matrix, a feature is added at a time, maximizing the model score. In sequential backward selection, starting from the full feature matrix, a feature is eliminated at a time, which minimally reduces the model MCC score. Sequential forward and backward selection were both applied to the feature matrix resulting from the covariance elimination step, and the features selected by both methods were combined to form the 18-dimensional final feature matrix.

Experimental methods

Peptide synthesis

Peptides were synthesized using the Fmoc (9-fluorenylmethyloxycarbonyl) Merrifield synthesis procedure[20] with a Symphony solid phase fully automated peptide synthesizer (Gyros Protein Technologies Inc., Tucson, AZ, USA). A 10-fold excess of Fmoc-protected amino acids (Gyros Protein Technologies Inc., Tucson, AZ, USA) dissolved in DMF (Dimethylformamide, Honeywell Specialty Chemicals, Seelze, Germany) was used relative to the Rink-amide-MBHA resin (Fmoc-protected Rink-amide-methylbenzhydrylamine resin, AAPPTec, Louisville, KY, USA) with 0.52 mmol g^{-1} loading and 100-200 mesh size. The amount of resin was calculated for a desired synthesis scale of 50 µmol. Coupling was performed with a ratio of 1:1:2 amino acid:HCTU:NMM (HCTU: 1H-benzotriazolium-5-chloro-1-[bi(dimethylamino)methylene] hexafluorophosphate (1-),3-oxide , AAPPTec, Louisville, KY, USA; NMM: 4-methyl-morpholine, Thermo Fisher Scientific, Waltham, MA, USA) in DMF. Fmoc deprotection was achieved with 20% v/v pyrrolidine (Thermo Fisher Acros Organics, Geel, Belgium) solution in DMF. Peptide cleavage from the resin was achieved with a TFA (2,2,2-trifluoroacetic acid, Thermo Fisher Scientific, Waltham, MA, USA) solution containing 2.5% v/v TIPS (triisopropylsilane, Sigma Aldrich, St. Louis, MO, USA) and 2.5% v/v distilled water.

Peptide purification

The peptides were precipitated using diisopropyl ether stabilized with 2,6-ditertbutyl-4-methylphenol (Merck KGaA, Darmstadt, Germany) and purified to >90% UV 190 nm on a preparative Prominence LCMS instrument (LC-20A, Shimadzu, Kyoto, Japan) using a reverse phase C18, 5 µm, 150 x 21 mm column (Macherey-Nagel GmbH & Co. KG, Düren, Germany), with a linear gradient of 5 to 70% acetonitrile (Merck KGaA, Darmstadt, Germany) over 25 min in water with 0.1% v/v formic acid (Sigma Aldrich, Steinheim, Germany) and a flow rate of 24.5 mL min^{-1}. The peptides were analyzed on a Prominence high performance liquid chromatography instrument (Shimadzu, Kyoto, Japan) using a reverse phase Nucleodur C18 HTec column (5 µm, 150 x 3 mm) (Macherey-Nagel GmbH & Co. KG, Düren, Germany), with a linear gradient of 5 to 70% acetonitrile in water with 0.1% formic acid over 25 min and a flow rate of 0.5 mL min^{-1}. Detection was performed by UV at a wavelength of 190 nm.

Circular dichroism

The circular dichroism (CD) spectra of peptides was measured with a Chirascan spectrometer (Applied Photophysics, UK). Peptide concentrations of 30 µm were measured in water and in a 1:1 v/v TFE:water mixture (TFE: 2,2,2-trifluoroethanol, 99.8% extra pure, AcrosOrganics, USA) using a 1 mm thick glass cuvette (Helma Analytics, Type No. 110-QS). CD was measured in a wavelength range from 180 to 260 nm with a step size of 1 nm. Triplicate measurements were averaged, the solvent baseline absorption was subtracted and the curves were smoothed with a window size of 4 in the Pro-Data Viewer software (Applied Photophysics, UK, version 4.2.15).

Cancer cell cultures

Michigan Cancer Foundation 7 (MCF7) human breast adenocarcinoma and A549 human adenocarcinoma alveolar basal epithelial cancer cells were provided by Prof. Dr. Cornelia Halin-Winter at the Institute of Pharmaceutical Sciences, ETH Zurich[21]. Cells were grown in Dulbecco's Modified Eagle Media (DMEM) containing 4.5 gL^{-1} D-glucose supplemented with

10% fetal bovine serum and 1% penicillin-streptomycin (Thermo Fisher Scientific, Waltham, MA, USA). The cultures were kept at 37°C in a 5% CO_2 atmosphere incubator (Thermo Fisher Scientific, Waltham, MA, USA) and split every two to three days to new culture flasks. Cells no older than 20 passages were used for experiments.

For splitting, the cell culture media was aspirated, the cells were washed with 0.01 M phosphate buffered saline (PBS, Gibco, Thermo Fisher Scientific, Waltham, MA, USA) and trypsin-EDTA (trypsin - ethylene diamine tetraacetic acid, Fisher scientific, UK) was added to the cell layer to detach them from the flask surface. After incubation for 2 min at 37°C, cells were resuspended with an equal volume of DMEM media and centrifuged at 310 g for 5 min at 4°C. After removal of the supernatant the cells were resuspended in DMEM cell media and split into new cell culture flasks at a 1/3 to 1/5 ratio.

Cell viability assay

Determination of the anticancer peptides half effective concentration (EC_{50}) was performed by a 3-(4,5-dimethylthiazol-2-yl)-2,5-diphenyltetrazolium bromide (MTT) assay, as described elsewhere[21]. Briefly, 10^4 cells were seeded in each well of a 96-well plate and left to adhere and grow for 24 h. For EC_{50} determination, peptides were dissolved in PBS with 1% dimethyl sulfoxide (DMSO, Fisher Scientific, UK) at an intermediate concentration of 1 mM. The peptides were then diluted in a 2-fold dilution series starting at 100 µM with DMEM culture media. Cells were incubated with the peptide solutions for 24 h. After this time, the cell media was removed and the cells were incubated for 1 h with a 0.5 mg mL^{-1} MTT solution to allow enough time for formazan crystal formation (Sigma Aldrich, Steinheim, Germany). The supernatant was removed and 100 µL of DMSO were added into each well to resuspend the formazan crystals. After manually shaking the plates to completely dissolve the crystals, the absorbance was measured at 540 nm in a Tecan Infinity M1000 spectrophotometer. The cell viability was determined based on the quantification of the color intensity in each culture well, considering the absorbance obtained by the control media without peptides as a 100% survival. The resultant sigmoidal curves were analyzed with the *drc*[22] R package using a log-logistic model with 3 fixed parameters to obtain the peptide concentration values that corresponded to 50% cell survival (EC_{50}).

Hemolysis assay

Human erythrocytes were freshly ordered at the Blutspende Zürich. Erythrocytes were washed 3 times with a 10 mm phosphate buffered saline (PBS, Gibco, Thermo Fisher Scientific, Waltham, MA, USA) solution centrifuging at 800 g for 10 min or until the supernatant appeared clear to remove the blood plasma. A 5% v/v erythrocytes : PBS solution was prepared for direct use. For HC_{50} determination, peptides were dissolved in PBS at an intermediate concentration of 1 mM. A dilution series of the peptide from 200 to 6.25 µM was plated in a round-bottomed 96-well plate in triplicates, with a volume of 50 µL per well. PBS buffer and a solution of 0.1% Triton-X-100 (Fisher Chemical, Loughborough, UK) in PBS were employed as negative and positive controls, respectively. 50 µL of the 5% erythrocyte solution were added in each well and the plates were incubated at 37°C for 1 h. After this time, 100 µL PBS were added to each well and plates were centrifuged at 800 g for 10 min. 100 µL of the supernatant were transferred to a flat-bottomed 96-well plate and the absorbance was measured at 540 nm in a Tecan Infinity M1000 spectrophotometer plate reader. The hemolysis percentage was calculated, considering the absorbance values of the Triton and PBS wells as 100% and 0% hemolysis, respectively.

The resultant sigmoidal curves were analyzed with the *drc*[22] R package using a log-logistic model with 3 fixed parameters to obtain the HC_{50} values.

Microfluidics chip experiments

Single cell ACP action was observed on MCF7 cells entrapped in a microfluidic chip following the previously described methodology[23]. For visualization, cells were stained with calcein-AM at a 1 μM concentration. The calcein-AM penetrates into the cytosol of the cells and it is converted there by intracellular esterases to a fluorescent form that remains encapsulated in the cytosol. After calcein staining, cells were incubated with a fluorescently-labelled anti-EpCAM (human CD326) antibody (Mouse IgG2b, *κ* Isotype Ctrl with Alexa Fluor 647 dye, Biolegend, San Diego, CA, USA) at a ratio of 5 μL per one million cells. Cells were incubated in the dark for 30 min. Afterwards, cells were twice with complete DMEM medium by centrifugation at 500 g for 5 min. Cells were stored until use at 37°C under constant rotation on a MACSmix tube rotator (12 rpm, Miltenyi Biotec, Bergisch Gladbach, Germany) for a maximum of 2 h. Prior to insertion to the chip, the cells were filtered with a 20 μm gravity filter (Partec GmbH, Jettingen-Scheppach, Germany) to remove large cell agglomerates.

NCI-60 screening procedure

The NCI-60 screening procedure was performed by the Developmental Therapeutics Program of the National Cancer Institute. The procedure was performed as previously described[24] in 96-well microtiter plates. Each peptide was tested in a 5-concentration assay, starting at 10^{-4} M and diluting 10-fold to the lowest 10^{-8} M concentration. The peptides were incubated for 48 h and the cell concentration was assayed by using the sulforhodamine B assay[25] and optical density measurements. The growth inhibition (GI50) value[24], measures the growth inhibitory power of the drug agent. The GI50 value is the concentration of the test drug for which the condition in Eq. S1 is met, where T is the optical density of the test well after a 48-h exposure to the peptide, T_0 the optical density before applying the peptide drug and C the control optical density of a well where no peptide was applied.

$$100\,x\,\frac{T - T_0}{C - T_0} = 50 \qquad \text{(Eq. S1)}$$

In vitro dose-response data

Dose-response data for peptides in Table 1

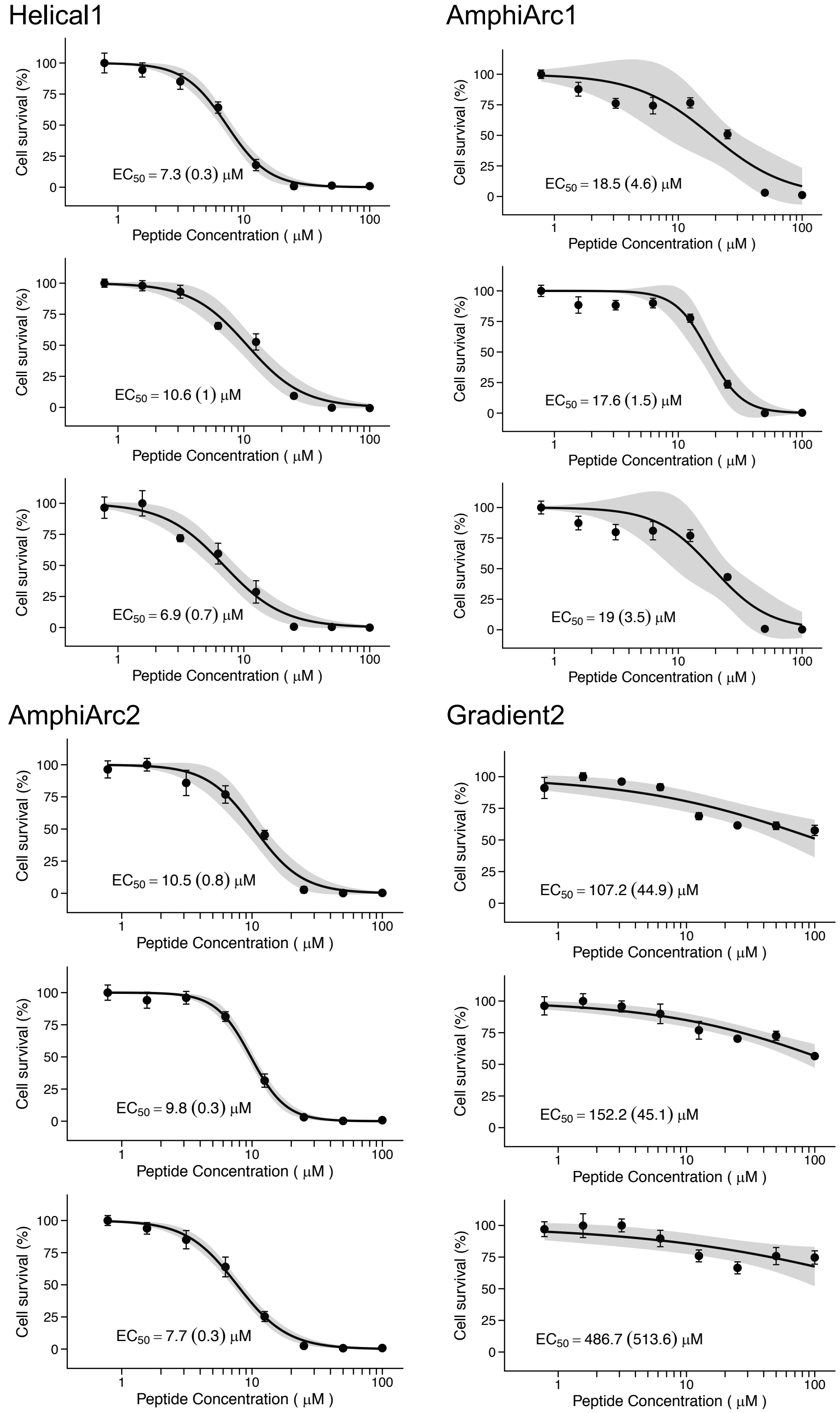

Figure S11. Dose-response curves for the peptides in Table 1 against the A549 lung cancer cell line. Experimental replicates are shown. Error bars denote technical replicates (*N* = 6). The continuous black line shows the fitting to a sigmoidal with a log-logistic model with 3 fixed parameters to obtain the EC_{50} values. The fitting curve uncertainty area is shown in light gray.

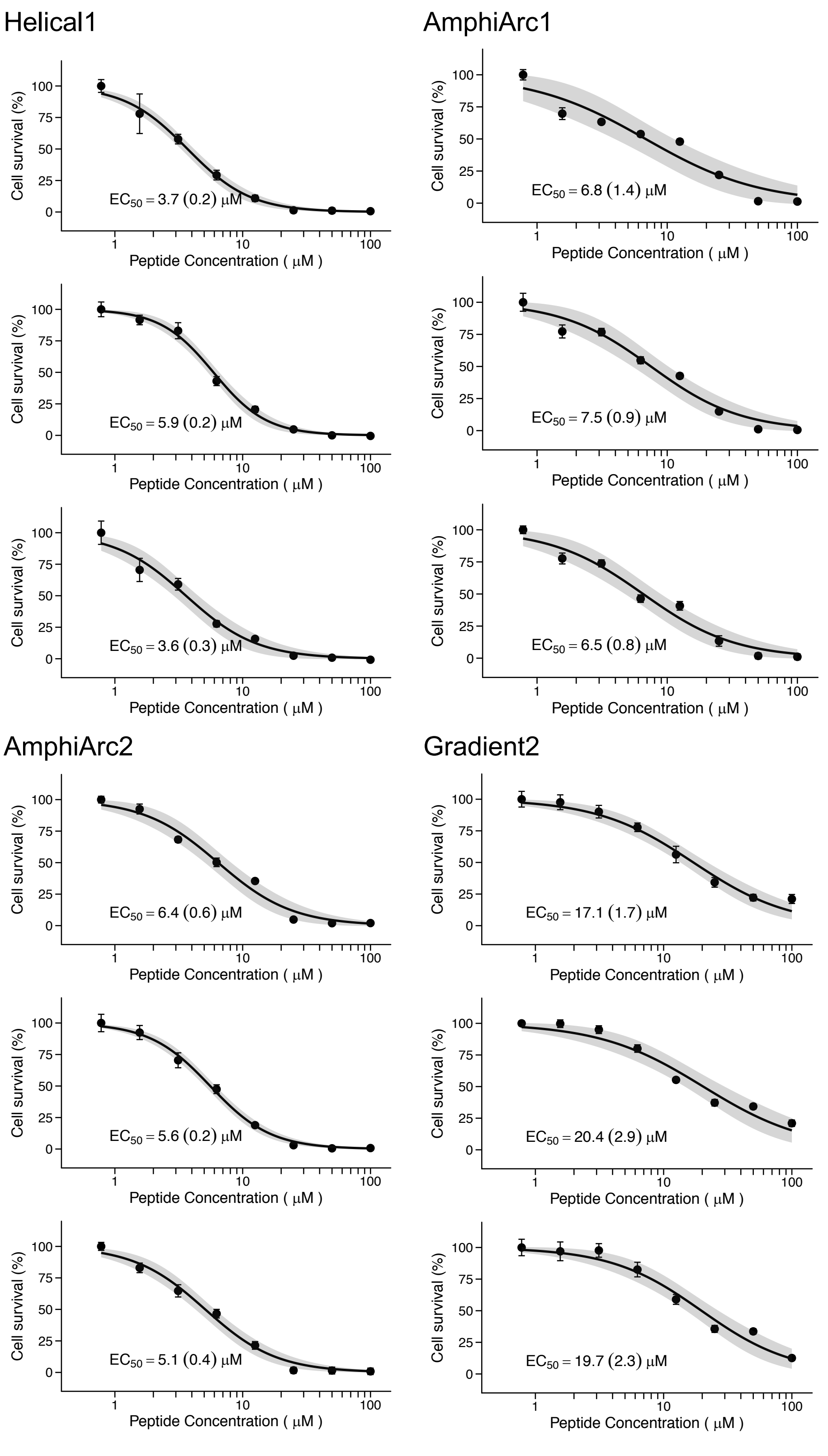

Figure S12. Dose-response curves for the peptides in Table 1 against the MCF7 breast cancer cell line. Experimental replicates are shown. Error bars denote technical replicates (*N* = 6). The continuous black line shows the fitting to a sigmoidal with a log-logistic model with 3 fixed parameters to obtain the EC_{50} values. The fitting curve uncertainty area is shown in light gray.

Dose-response data for the SME offspring

Table S2. Peptide sequences, molecular weight (MW), and activity on the A549 and MCF7 cancer cells (EC_{50}, *mean* ± *sd*), the HDMEC primary cell line (EC_{50}, *mean* ± *sd*), and hemolysis on human erythrocytes (HC_{50}, *mean* ± *sd*).

**Name**	**Sequence**[a]	**MW Da**	**A549 EC_{50} /µM**	**MCF7 EC_{50} /µM**	**HDMEC EC_{50} /µM**	**Eryhtr. HC_{50} /µM**
Parent	KIFKKFKTIIKKVWRIFGRF	2581.6	9 ± 2	5.7 ± 0.7	3.3 ± 0.2	16 ± 2
Off1	RVFKKFHPIYHRVWRLWGHL	2673.5	49 ± 2	27.4 ± 0.5	56 ± 7	> 200
Off2	HIIKKIRTWYRKAWHVLGKV	2530.5	17.0 ± 0.6	10 ± 2	32 ± 3	> 200
Off3	KLYKKFRHILKKVWHYVGKI	2582.6	14 ± 3	10 ± 1	10 ± 3	29 ± 3
Off4	KYIRHLKTWFKKVFKLIGEV	2531.5	15.0 ± 0.1	11 ± 2	7.1 ± 0.1	20 ± 3
Off5	KIFKKFKDFLKKIFQYLGKV	2516.5	17.4 ± 0.1	12.4 ± 0.4	10.1 ± 0.2	15 ± 2
Off6	HIFKKVKTYWKKLFRILGRF	2606.6	18 ± 2	12 ± 3	5.0 ± 0.7	16 ± 2
Off7	RLIKRLKTFVRKTWKWVGHF	2597.6	52 ± 2	39 ± 3	12 ± 3	95 ± 7
Off8	RFLKHFKTVYKRYWKVLGRL	2636.6	17 ± 4	11.9 ± 0.1	14.3 ± 0.2	144 ± 4
Off9	KLIHRLKTVFKKVWHFLGHL	2498.5	22 ± 4	18.9 ± 0.2	22 ± 3	41 ± 3
Off10	HRIRQLKTTIKKFWEIWPKI	2619.6	83 ± 11	56 ± 5	58.1 ± 0.3	> 200
Off2.1	QVFKKFRPFYRRPWELFGKL	2640.5	> 100	91 ± 2	173 ± 1	> 200
Off2.2	QIIKKIRTWYRKAWHVLGKV	2521.5	20 ± 1	11.6 ± 0.8	42 ± 7	> 200
Off2.3	TLYKKFRHFLKKPWHVVGKI	2523.5	> 100	65 ± 3	167 ± 13	> 200
Off2.4	HYIRHLKTWFHKPFKLIGKV	2546.5	> 100	> 100	169 ± 28	> 200
Off2.5	KIFKKFKDWFKKAFHVLGKV	2492.5	14.8 ± 0.7	10.4 ± 0.8	22 ± 1	> 100
Off2.6	QIFKKVKTWYKKAFQILGRL	2493.5	12.4 ± 0.1	9.3 ± 0.2	21 ± 2	> 100
Off2.7	RLIKRIKTWYRKAWKVVGKF	2574.6	15 ± 1	11.2 ± 0.7	23 ± 5	> 200
Off2.8	RFLKHLHTYYERAWHVIGHL	2574.4	> 100	> 100	2 ± 2	> 200
Off2.9	HLIHRLHTYWHKPWHYLGKL	2633.4	> 100	> 100	135 ± 2	> 200
Off2.10	QRIRQLHTWIKKAWHIWPKI	2636.5	40 ± 2	28.6 ± 0.1	39.7 ± 0.3	> 200
Off2.2.1	QVVKKIRTWYHKAWHVLGKV	2476.0	> 100	76 ± 15	96 ± 7	> 200
Off2.2.2	QLIHKIRTWYRKAWHVLGKV	2532.1	20 ± 2	13 ± 1	28 ± 3	> 200
Off2.2.3	HLLKKWRTWLRKAWHIVGKV	2555.2	9 ± 1	7 ± 1	6.1 ± 0.5	59 ± 6
Off2.2.4	QVLKKVRTWYRKAFHVIGKV	2456.0	> 100	68 ± 5	> 100	> 200
Off2.2.5	QLLKKVRTWYRKAWHLYGKV	2573.1	29.3 ± 0.9	16 ± 2	27.2 ± 0.5	> 200
Off2.2.6	QLIKKLRTWYRKAWHVLGKL	2537.1	10.6 ± 0.8	6.5 ± 0.2	9 ± 1	> 200
Off2.2.7	QIIKKVRTWIKKAWHLIGKI	2459.1	12 ± 2	8 ± 1	17 ± 1	> 100
Off2.2.8	QYLRRVRTWLRRAWHILGKV	2607.2	16.5 ± 0.2	11.0 ± 0.2	10.3 ± 0.3	21 ± 1
Off2.2.9	QLLKKIRTWYRKAWHVYGKV	2573.1	28 ± 3	13 ± 1	21 ± 2	> 200
Off2.2.10	QIYKKYRTWYRKAWKVLGKV	2614.2	36 ± 7	16.9 ± 0.8	71 ± 12	> 200

[a]All peptides were synthesized with C-terminal amidation.

Dose-response data on the NCI-60 cell panel

Table S3. Cellular growth inhibition of 60 cell lines in the NCI-60 cancer cell test for the parent (AmphiArc2), Off2 and Off2.2.10 peptides. The averaged peptide activity for each individual cell line and the averaged activity for each cancer type tested is shown as the logarithm of the half growth inhibitory concentration (GI50), the molar concentration of peptide needed to inhibit half of the normal cancer cell growth.

	Cell line	Parent Log GI (M)		Off 2 Log GI (M)		Off 2.2.10 Log GI (M)	
**Leukemia**	CCRF-CEM	-5.60	-5.50	-5.43	-5.17	-5.38	-5.33
	HL-60	-5.28		-5.37		-5.55	
	K-562	-5.55		-5.18		-5.49	
	MOLT-4	-5.51		-4.85		-4.93	
	RPMI-8226	-5.51		-5.06		-5.16	
	SR	-5.57		-5.15		-5.45	
**Lung**	A549/ATCC	-5.60	-5.65	-5.26	-5.37	-4.90	-5.24
	EKVX	-5.73		-5.63		-5.52	
	HOP-62	-5.62		-5.34		-5.29	
	HOP-92	-5.70		-5.68		-5.71	
	NCI-H226	-5.59		-5.38		-4.91	
	NCI-H23	-5.61		-5.31		-5.40	
	NCI-H322M	-5.73		-5.30		-5.34	
	NCI-H460	-5.62		-5.48		-5.49	
	NCI-H522	-5.63		-4.96		-4.59	
**Colon**	COLO 205	-5.58	-5.57	-5.68	-5.24	-5.37	-5.10
	HCC-2998	-5.58		-5.41		-4.76	
	HCT-116	-5.49		-4.76		-4.97	
	HCT-15	-5.58		-5.37		-5.53	
	HT29	-5.53		-4.83		-4.68	
	KM12	-5.63		-5.60		-5.53	
	SW-620	-5.59		-5.02		-4.89	
**CNS[a]**	SF-268	-5.68	-5.63	-5.46	-5.21	-5.40	-5.22
	SF-295	-5.69		-5.32		-5.33	
	SF-539	-5.72		-5.67		-5.66	
	SNB-19	-5.58		-4.74		-4.76	
	SNB-75	-5.60		-4.92		-5.22	
	U251	-5.52		-5.14		-4.92	
**Melanoma**	LOX IMVI	-5.53	-5.64	-4.66	-5.33	-4.62	-5.25
	MALME-3M	-5.89		-5.64		-5.66	
	M14	-5.62		-5.38		-5.51	
	MDA-MB-435	-5.63		-5.29		-5.24	
	SK-MEL-2	-5.58		-5.28		-4.78	

	SK-MEL-28	-5.66		-5.58		-5.31	
	SK-MEL-5	-5.64		-5.40		-5.55	
	UACC-257	-5.54		-5.07		-4.86	
	UACC-62	-5.69		-5.69		-5.68	
**Ovarian**	IGVROV1	-5.68	-5.60	-5.36	-5.17	-5.51	-5.24
	OVCAR-3	-5.67		-4.98		-5.37	
	OVCAR-4	-5.67		-4.89		-5.04	
	OVCAR-5	-5.68		-5.21		-5.06	
	OVCAR-8	-5.48		-5.32		-5.42	
	NCI/ADR-RES	-5.53		-5.23		-5.32	
	SK-OV-3	-5.50		-5.20		-4.95	
**Renal**	786-0	-5.53	-5.67	-5.47	-5.28	-5.52	-5.22
	ACHN	-5.71		-4.88		-4.92	
	CAKI-1	-5.71		-5.48		-5.41	
	RXF-393	-5.68		-5.37		-5.34	
	SN12C	-5.69		-5.53		-5.45	
	TK-10	-5.65		-5.49		-5.20	
	UO-31	-5.74		-4.73		-4.72	
**Prostate**	PC-3	-5.66	-5.72	-5.55	-5.46	-5.42	-5.44
	DU-145	-5.77		-5.37		-5.45	
**Breast**	MCF7	-5.52	-5.65	-5.69	-5.36	-5.73	-5.43
	MDA-MB-231/ATCC	-5.72		-5.19		-5.36	
	HS-578T	-5.65		-5.45		-5.59	
	BT-549	-5.45		-4.89		-5.27	
	T-47D	-5.66		-5.72		-5.69	
	MDA-MB-468	-5.88		-5.19		-4.96	

[a]CNS: central nervous system.

Supplementary files

Supplementary Video SV1

Supplementary video (*.avi format) shows the membranolysis of a single MCF7 cell entrapped in a microfuidic chip chamber after exposure to the AmphiArc2 peptide at a concentration of 50 μM. The cells were stained with calcein-AM and fluorescently-labelled anti-EpCAM (human CD326) to visualize their cellular membrane. The loss of the cytosolic fluorescence corresponds to a permeabilization of the cell membrane. Imaging was performed at 10 Hz with a 100x oil immersion objective and an Andor iXON Ultra EMCCD camera with an electron-multiplying gain of 100 and 50 ms exposure.

Training data set

Data set employed for training the SVM model (*.csv format). The column sequence contains the peptide sequences in a 1-letter amino acid code. The column class denotes if the peptide was used as positive (active) class (1) for training, or as negative (inactive) class (0).

References

1. Tyagi, A. *et al.* In silico models for designing and discovering novel anticancer peptides. *Sci. Rep.* **3,** 2984 (2013).
2. Waghu, F. H. *et al.* CAMP: Collection of sequences and structures of antimicrobial peptides. *Nucleic Acids Res.* **42,** 1154–1158 (2014).
3. Novkovic, M., Simunic, J., Bojovic, V., Tossi, A. & Juretic, D. DADP: the database of anuran defense peptides. *Bioinformatics* **28,** 1406–1407 (2012).
4. Wang, G., Li, X. & Wang, Z. APD3: the antimicrobial peptide database as a tool for research and education. *Nucleic Acids Res.* **44,** 1087–1093 (2016).
5. Bateman, A. *et al.* UniProt: The universal protein knowledgebase. *Nucleic Acids Res.* **45,** D158–D169 (2017).
6. Chen, W., Ding, H., Feng, P., Lin, H. & Chou, K. iACP: a sequence-based tool for identifying anticancer peptides. *Oncotarget* **7,** 16895–16909 (2016).
7. Manavalan, B. *et al.* MLACP: machine-learning-based prediction of anticancer peptides. *Oncotarget* **8,** 77121–77136 (2017).
8. Eisenberg, D., Weiss, R. M. & Terwilliger, T. C. The helical hydrophobic moment: a measure of the amphiphilicity of a helix. *Nature* **299,** 371–374 (1982).
9. Müller, A. T., Gabernet, G., Hiss, J. A. & Schneider, G. modlAMP: Python for antimicrobial peptides. *Bioinformatics* (2017). doi:10.1093/bioinformatics/btx285
10. Tyagi, A. *et al.* CancerPPD: a database of anticancer peptides and proteins. *Nucleic Acids Res.* **43,** 837–843 (2015).
11. Berman, H. M. The Protein Data Bank. *Nucleic Acids Res.* **28,** 235–242 (2000).
12. R Development Core Team. *R: A language and environment for statistical computing. R Foundation for Statistical Computing, Vienna, Austria.* (2011). at <http://www.r-project.org.>
13. Grant, B. J., Rodrigues, A. P. C., ElSawy, K. M., McCammon, J. A. & Caves, L. S. D. Bio3d: an R package for the comparative analysis of protein structures. *Bioinformatics* **22,** 2695–2696 (2006).
14. Charif, D. & Lobry, J. R. in *Structural approaches to sequence evolution: Molecules,*

networks, populations (ed. Vendruscolo, U. B. and M. P. and H. E. R. and M.) 207–232 (Springer Verlag, 2007).
15. Li, W. & Godzik, A. Cd-hit: a fast program for clustering and comparing large sets of protein or nucleotide sequences. *Bioinformatics* **22,** 1658–1659 (2006).
16. Koch, C. P. *et al.* Scrutinizing MHC-I binding peptides and their limits of variation. *PLoS Comput. Biol.* **9,** e1003088 (2013).
17. Moreau, G. & Broto, P. Autocorrelation of a topological structure: a new molecular descriptor. *Nouv. J. Chim.* **4,** 359–360 (1980).
18. Pedregosa, F. *et al.* Scikit-learn: machine learning in Python. *J. Mach. Learn. Res.* **12,** 2825–2830 (2011).
19. Jolliffe, I. T. *Principal Component Analysis*. (Springer New York, 1986). doi:10.1007/978-1-4757-1904-8
20. Merrifield, R. B. Solid Phase Peptide Synthesis. I. The Synthesis of a Tetrapeptide. *J. Am. Chem. Soc.* **85,** 2149–2154 (1963).
21. Lin, Y.-C. *et al.* Multidimensional design of anticancer peptides. *Angew. Chemie Int. Ed.* **54,** 10370–10374 (2015).
22. Ritz, C., Baty, F., Streibig, J. C. & Gerhard, D. Dose-Response Analysis Using R. *PLoS One* **10,** e0146021 (2015).
23. Armbrecht, L. *et al.* Characterisation of anticancer peptides at the single-cell level. *Lab Chip* **17,** 2933–2940 (2017).
24. Monks, a *et al.* Feasibility of a high-flux anticancer drug screen using a diverse panel of cultured human tumor cell lines. *J. Natl. Cancer Inst.* **83,** 757–766 (1991).
25. Rubinstein, L. V *et al.* Comparison of in vitro anticancer-drug-screening data generated with a tetrazolium assay versus a protein assay against a diverse panel of human tumor cell lines. *J. Natl. Cancer Inst.* **82,** 1113–8 (1990).
